# Zinc Coordination by Thymosin β4: Structural Determinants and Functional Implications

**DOI:** 10.3390/ijms27041740

**Published:** 2026-02-11

**Authors:** Joanna Izabela Lachowicz, Terenzio Congiu, Andrea Salis, Flaminia Cesare Marincola

**Affiliations:** 1 Department of Environmental Health, Occupational Medicine and Epidemiology, Wroclaw Medical University, Mikulicza-Radeckiego 7, 50-368 Wroclaw, PL, Poland; 2 Department of Medical Science and Public Health, University of Cagliari, Cittadella Universitaria, 09042 Monserrato, CA, Italy; terenzio.congiu@unica.it; 3 Department of Chemical and Geological Sciences, University of Cagliari and CSGI, Cittadella Universitaria, 09042 Monserrato, CA, Italy; asalis@unica.it (A.S.); flaminia@unica.it (F.C.M.)

**Keywords:** thymosin β4 (Tβ4), electrospray ionization mass spectrometry (ESI-MS), scanning electron microscopy (SEM), zinc (Zn)

## Abstract

Thymosin β4 (Tβ4) is a highly acidic, intrinsically disordered 43-amino-acid peptide with diverse biological functions, yet its interactions with metal ions remain poorly understood. In this study, we provide the first experimental demonstration that Tβ4 forms discrete Zn^2+^-bound adducts and undergoes Zn^2+^-induced aggregation under physiological pH conditions. Combining zeta potential analysis, dynamic light scattering (DLS), electrospray ionization mass spectrometry (ESI-MS), nuclear magnetic resonance (NMR) spectroscopy, and scanning electron microscopy with elemental mapping (SEM/EDS), we show that Zn(II) binding progressively neutralizes Tβ4’s negative surface charge and triggers a sharp aggregation transition. ESI-MS unambiguously identifies Tβ4/Zn(II) complexes of peptide-to-zinc molar ratio 1:3, while DLS and SEM reveal the formation of compact, low-solubility supramolecular assemblies. NMR measurements support a metal-induced aggregation, confirming the absence of folding upon Zn(II) binding. By quantitatively comparing the experimentally determined critical aggregation concentration with physiologically observed extracellular Zn(II) ranges, we demonstrate that aggregation is unlikely in plasma or basal interstitial environments but may become feasible in Zn-rich microdomains, such as the synaptic cleft, where transient Zn(II) levels can exceed 1 μM. These findings introduce a previously unrecognized dimension of Tβ4 chemistry and suggest that a Zn(II)-mediated supramolecular assembly of Tβ4 could influence peptide behavior in neurological or inflammatory conditions characterized by elevated extracellular Zn(II). This work establishes a foundational biochemical framework for future studies aimed at elucidating the biological implications of Tβ4/Zn(II) complexation and aggregation in vivo.

## 1. Introduction

**Figure 1:**
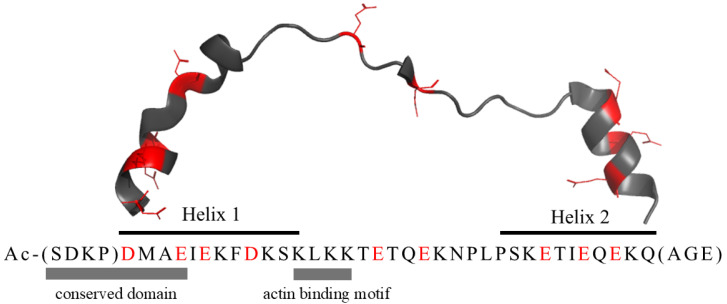
X-ray structure of Tβ4 extracted from the complex with actin (PDB 4PL7). Asp and Glu residues are marked in red. Amino acids in parentheses were not defined by the X-ray structure.

Timbetasin (Thymosin β4, Tβ4) is a peptide encoded by the TMSB4X (synonyms: TΒ4X, THYB4, TMSB4) gene and composed of 43 amino acids (molecular weight 4982 Da; Figure 1). The primary sequence of Tβ4 is highly conserved across a wide range of species, including mammals, birds, fish, and even invertebrates [1,2]. This conservation underscores its fundamental biological importance. It has an acidic isoelectric point (pI = 5.1) due to the presence of 11 amino acids with carboxylic acid groups in their side chains (Glu (E) and Asp (D), signed in red in Figure 1). The N-terminal acetylation of Tβ4 is a well-established post-translational modification catalyzed by NAT enzymes in vivo (UniProt Q95274).

**Figure 2:**
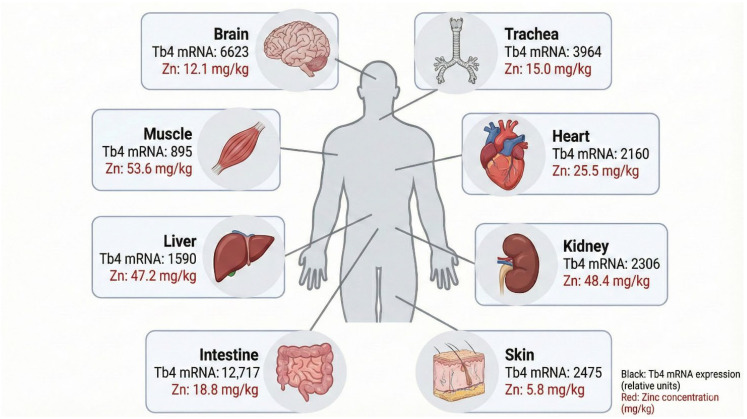
Tβ4 mRNA expression shown in black (based on data from Su et al. [9]) and zinc(II) ion concentrations shown in red (according to the *Report of the Task Group on Reference Man*, ICRP Publication 23 [10]) in selected human tissues.

Among different thymic peptides (Thymosin alpha 1, thymulin, and thymopoietin), Tβ4 is one of the most important. Tβ4 is also found in the spleen, peritoneal macrophages, brain, lungs, liver, heart muscle, kidneys, platelets, and leukocytes [3,4] (Figure 2). Additionally, it is present in body fluids, namely tears, saliva, cerebrospinal fluid, and serous exudate from wounds [5,6,7]. Human serum levels of Tβ4 range from 3 to 82 ng/mL [8]. Of note, newborn cord serum levels of Tβ4 are lower than in adults [8]. Another study showed that Tβ4 concentrations range from 0.5 to 7 µg/mL in tears and 0.2–3.6 µg/mL in saliva. In both fluids, Tβ4 concentration varied with age and appeared to peak at ages 25–35 years [7].

The acetylated N-end of the Tβ4 peptide is composed of the hemoregulatory peptide AcSDKP, which is a significant inhibitor of bone marrow-derived stem cell differentiation by enabling the entry of hematopoietic pluripotent stem cells into the S-phase [11]. Moreover, AcSDKP has potent anti-fibrotic effects in organs like the heart, kidneys, lungs, and liver. It inhibits TGF-β/Smad signaling, reducing fibroblast activation and collagen deposition [12], and reduces macrophage infiltration, stabilizes endothelial cells, and promotes angiogenesis during tissue repair. The cleavage of AcSDKP from Tβ4 occurs in specific tissues and under particular physiological conditions, primarily involving angiotensin-converting enzyme (ACE) and prolyl oligopeptidase (POP) [12]. In liver injury models, Tβ4-derived AcSDKP has shown anti-fibrotic effects, suggesting that cleavage occurs in hepatic tissues under stress or inflammation [13], while in the heart, especially the epicardium, Tβ4 is cleaved to release AcSDKP during cardiac development and post-injury repair [14].

Tβ4 has a wide range of biological activity. It is involved in G-actin fibers [15], cytoskeletal and lipid binding, ligase and molecular sequestering activity. Thus, Tβ4 takes part in the mitotic cell cycle, mitochondrial organization, microtubule-based movement and cell differentiation processes. In consequence, Tβ4 plays an important role in tissue regeneration and wound healing by stimulating the migration of keratinocytes and endothelial cells [5,16] and increasing angiogenesis [17] by recruiting endothelial progenitor cells (EPCs) and promoting their differentiation [18]. Ildiko Bock-Marquette et al. [19] showed that Tβ4 promotes the migration and survival of cardiac cells during the growth phase of the organism and can change the morphology of the epicardium in adults and activate epicardial progenitors independently of hypoxia-related damage.

Tβ4 inhibits a series of processes such as the production of key inflammatory cytokines by downregulating the NFkB pathway [20,21] and apoptosis [22,23]. In addition, it influences stem cell differentiation [24], regulates metalloproteinase activity [25] and protects cells against oxidative stress by reducing the generation of reactive oxygen species (ROS) [26]. Our previous study has revealed an involvement of Tβ4 in ferroptosis, an iron-dependent process of programmed cell death [27], where it functions as an endogenous iron chelator, regulating free iron ion homeostasis. Recent studies [28] have shown that Tβ4 slows down the aging process by inhibiting age-promoting processes, such as telomerase activity and the serine/threonine protein kinase Akt cascade [29].

Cross-Linking–Mass Spectrometry (CLMS), a technique that detects an interaction between two proteins using chemically reactive or photo-activatable cross-linking reagents to covalently link amino acids in close proximity, showed high affinity of Tβ4 to zinc finger CCCH-type. In addition, affinity-capture MS (a technique that identified protein interaction, which is inferred when a bait protein is affinity captured from cell extracts by either polyclonal antibody or epitope tag followed by MS analysis of the associated partners) revealed high affinity of Tβ4 to zinc finger protein 526 [30].

Zinc is the second most concentrated trace element in the human body. Its total content in the healthy adult body changes between 1.4 and 2.3 g, and it is abundant in muscles and bones (~85% of the total zinc amount). However, the human brain contains the highest concentration of zinc among all organs, with levels exceeding those found in the liver and serum by up to tenfold. This elevated concentration is particularly notable in regions such as the hippocampus, cerebral cortex, and olfactory bulb, where zinc plays a critical role in synaptic transmission and plasticity [31]. Zinc is stored in synaptic vesicles of glutamatergic neurons and released into the synaptic cleft during neuronal activity. This release allows zinc to modulate neurotransmitter receptors (e.g., N-methyl-D-aspartate receptor (NMDA), α-amino-3-hydroxy-5-methyl-4-isoxazolepropionic acid (AMPA), γ-aminobutyric acid (GABA) receptor), ion channels, and intracellular signaling pathways. The high local concentration of zinc in the brain supports its function as a neuromodulator and second messenger, influencing learning, memory, and neuroprotection. The high concentration of zinc in the brain suggests a potential interaction between Tβ4 and zinc ions in neural tissues. Tβ4 is expressed in various brain regions and may participate in neuroprotective and regenerative processes [2,32]. While the cleavage of AcSDKP is less prominent in the brain, the intact Tβ4 peptide may still play a role in zinc homeostasis and signaling within neural environments.

Zinc liver content depends on gestational age and decreases in the postnatal period [33]. In plasma, 0.1% of total body zinc is bound to albumin [34], α-2-micro-globulin and transferrin [35]. Next to iron, copper, manganese, and selenium, zinc is an essential metal ion in human physiology. Unlike other transition metals such as iron or copper, zinc does not participate in redox reactions under physiological conditions, due to its stable d10 electronic configuration. While zinc clearly has redox potential in chemical systems (e.g., batteries), its biological role is primarily structural and catalytic, rather than redox-active. Zinc is a key structural and catalytic component in approximately 3000 human proteins, accounting for nearly 10% of the proteome, including numerous enzymes and transcription factors [36].

While intracellular free Zn(II) concentrations are tightly buffered and typically remain in the sub-nanomolar range [37], transient elevations in local zinc levels—particularly in extracellular fluids (e.g., tears, wound exudates) [38] or in zinc-rich tissues such as the brain and spleen—may permit interactions with lower-affinity ligands [39]. Tβ4 is known to be secreted and is present in extracellular compartments, where zinc concentrations can fluctuate due to synaptic activity, inflammation, or oxidative stress [38,39]. These conditions may allow transient Zn(II)–Tβ4 interactions that could influence peptide conformation, aggregation, or biological activity.

In 1990 the Tβ4’s structure in water was investigated [40] by NMR (however, it was not deposited in Protein Database) and CD spectroscopy, revealing the absence of a stable, well-defined conformation. Nevertheless, the addition of Trifluoroethanol (TFE), a known foster of helical structure formation, led to the formation of a stable tertiary structure with two α-helices, previously on the X-ray structure (Figure 1, from Pro4 to Lys16 and from Ser30 to Ala40). As X-ray structural data for free Tβ4 is unavailable, Figure 1 displays the X-ray structure of Tβ4 derived from its complex with actin (PDB: 4PL7). The 3D structure of Tβ4 (Figure 1) exhibits the same structural characteristics as those identified through NMR analysis in 1990.

To better characterize the formation of Zn(II)–Tβ4 complexes, we employed a suite of analytical and thermodynamic methods, including zeta potential analysis, dynamic light scattering (DLS), electrospray ionization mass spectrometry (ESI-MS), scanning electron microscopy (SEM), and nuclear magnetic resonance (NMR) spectroscopy. Collectively, these techniques indicate that Zn(II) associates with Tβ4 to generate stable, poorly soluble complexes. The potential biochemical relevance of this interaction is explored.

## 2. Results

### 2.1. I-TASSER

To complement the experimental characterization of Thymosin β4 (Tβ4), we performed ab initio structural modeling with the I-TASSER server. This analysis was intended exclusively to assess global foldability and intrinsic flexibility, not to infer function or metal-binding properties, an important limitation for intrinsically disordered peptides (IDPs) such as Tβ4. The full output is provided in the Supplementary Materials (Figures S1 and S2, Tables S1–S4).

The threading step of I-TASSER identified only low-identity structural templates, with sequence identity values predominantly below 20% and normalized Z-scores close to 1.0 (Table S1). Such scores indicate weak alignment significance, consistent with the lack of a stable globular fold. The predicted secondary structure profile showed a coil-dominated sequence with scattered helical segments (Table S1, top row), while the predicted normalized B-factor values were uniformly elevated across the peptide (Figure S1), indicating high flexibility and conformational heterogeneity.

Five structural models were generated by clustering simulation decoys (Table S2). All models exhibited low C-scores (ranging from −1.24 to −5.00) and large estimated RMSD values, reflecting poor convergence and the absence of a dominant fold within the ensemble. The graphical representations (Figure S2) further illustrate the lack of a conserved tertiary structure among the predicted models, a hallmark of IDPs.

Subsequent TM-align structural comparison of the top model (Table S3) produced moderate TM-scores (~0.60) but extremely low sequence identity (<10%), implying only superficial geometric similarity to unrelated proteins—a well-recognized artifact of modeling highly flexible sequences. Finally, although automated COFACTOR/COACH annotations (Table S4) list putative ligand-binding and functional predictions derived from template structures, these outputs are not biologically interpretable for Tβ4 due to the low model confidence and the peptide’s disordered nature. They are included solely for transparency.

Overall, the I-TASSER results are fully consistent with Tβ4 behaving as an intrinsically disordered peptide lacking a stable three-dimensional fold, reinforcing the need for experimental methods (NMR, DLS, MS) to probe its behavior upon Zn(II) interaction.

### 2.2. Zeta Potential and Dynamic Light Scattering (DLS) Analysis

Zeta potential measurements were employed to assess the colloidal stability and surface charge characteristics of the emerging Tβ4/Zn(II) aggregates. Because electrostatic interactions strongly influence peptide–metal association, charge screening, and subsequent aggregation behavior, monitoring zeta potential provides a sensitive indicator of complex formation and particle destabilization in aqueous environments [41]. A shift toward less negative (or near-neutral) zeta potential values typically reflects reduced electrostatic repulsion, favoring aggregate growth and precipitation, which is consistent with the formation of low-solubility Zn(II)–peptide assemblies [42]. Zeta potential analysis therefore offered a quantitative descriptor of the aggregation state of Tβ4 upon Zn(II) coordination and complemented structural techniques such as DLS, MS, and NMR.

**Figure 3:**
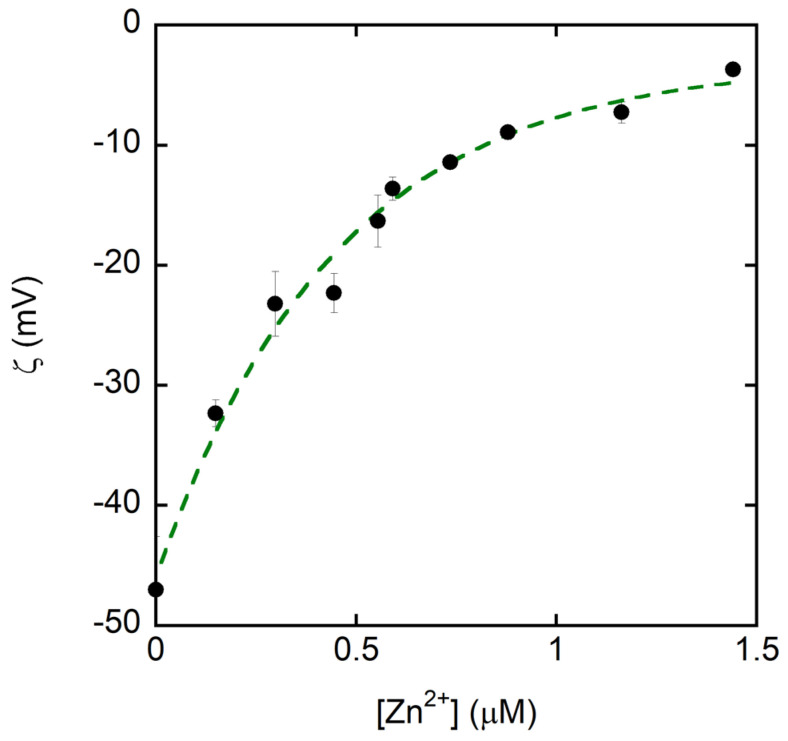
Zeta potential (ζ) of the peptide as a function of Zn(II) concentration. Increasing Zn(II) produces a progressive shift in ζ from ~−48 mV (0 μM Zn^2+^) toward near-neutral values (≈−5 mV) at ~1.5 μM Zn(II), consistent with Zn(II)-dependent charge compensation/association. Measurements were performed in salt-free, unbuffered solutions; the pH was monitored throughout the titration and remained between 7.48 and 7.74. Data are shown as mean ± SD (error bars) from replicate measurements; the dashed line is a guide for the eye.

Zeta potential measurements (Figure 3) reveal a monotonic shift from approximately −48 mV for the free peptide to near-neutral values (≈−5 mV) at around 1.5 μM Zn(II), indicating progressive charge neutralization upon metal addition. Because the experiments were conducted in unbuffered, salt-free solutions, the ζ trend is interpreted qualitatively as evidence of Zn(II)-dependent peptide association rather than a quantitative measure of binding affinity. The pH remained stable throughout the titration (7.48–7.74), ruling out pH drift as the cause of the observed ζ changes.

The free peptide displays a negative ζ potential, consistent with its abundance of deprotonated acidic residues at physiological pH. Incremental Zn(II) addition progressively reduces the ζ potential through coordination with carboxylate groups, weakening electrostatic repulsion as the peptide-to-metal ratio approaches unity.

Dynamic light scattering (DLS) was employed to characterize the diffusion coefficient of the Zn(II)–Tβ4 aggregates in solution. Because metal–peptide coordination often leads to changes in particle diameter, polydispersity, and aggregation kinetics, DLS provides a sensitive, non-destructive method to monitor the evolution of particle size during complex formation. Bhattacharjee et al. [41] emphasizes that DLS is one of the most widely used techniques for assessing nanoparticle size in colloidal systems due to its simplicity, reproducibility, and ability to measure hydrodynamic radii under near-physiological conditions. This makes DLS particularly suitable for detecting the transition from monomeric peptide species to higher-order Zn(II)–Tβ4 assemblies in aqueous buffers.

**Figure 4:**
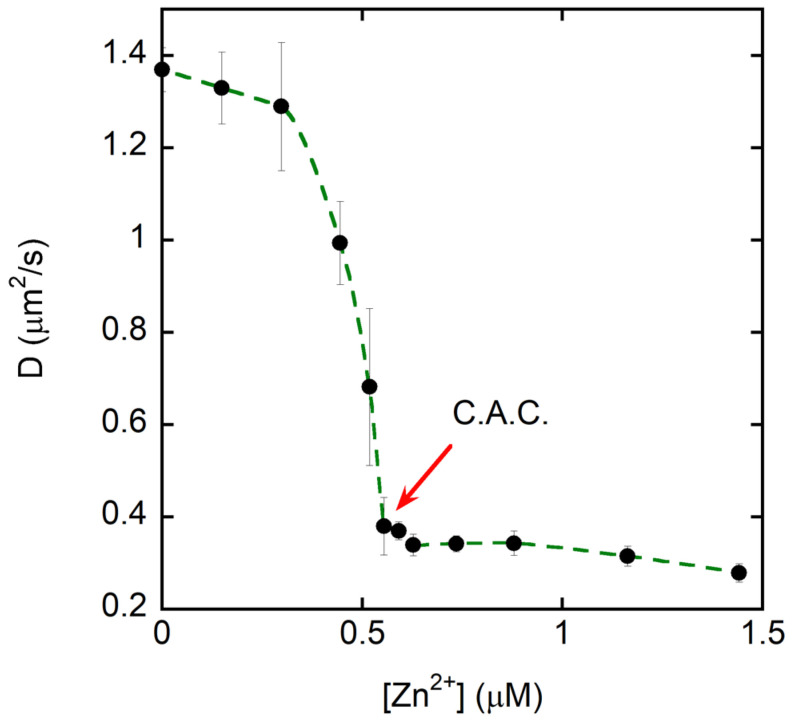
Dynamic light scattering (DLS) measurements of the translational diffusion coefficient (D) of the peptide as a function of Zn(II) concentration. D decreases sharply at ~0.5–0.6 μM Zn(II), indicating the onset of formation of larger species and defining a critical aggregation concentration (C.A.C.). Above the C.A.C., D remains low and weakly dependent on Zn(II), consistent with a predominant population of slow-diffusing (larger) assemblies. Data are shown as mean ± SD (error bars) from replicate measurements; the dashed line is a guide for the eye.

Moreover, changes in the diffusion coefficient obtained from DLS measurements complement the zeta potential data by providing an orthogonal indicator of aggregation. As noted by Honary and Zahir [42], particle size and surface charge are the two major physical parameters governing colloidal stability and aggregation in nanoscale systems; thus, combining DLS with zeta potential yields a more complete physicochemical picture of Zn(II)–Tβ4 association. DLS experiments (Figure 4) were performed to monitor variations in the diffusion coefficient of Tβ4 as a function of Zn(II) concentration. In the absence of Zn(II), the peptide exhibited diffusion behavior consistent with small, monomeric or minimally associated species, in line with its intrinsic disorder and high solubility at physiological pH. Upon Zn(II) titration, the DLS intensity profiles revealed the progressive appearance of slowly diffusing species, indicative of early metal-induced association. As the peptide-to-metal molar ratio approached 0.9 ([Zn^2+^] = 0.5 µM), the diffusion coefficient dropped sharply, reflecting the sudden formation of large aggregates. At this critical point, corresponding to the critical aggregation concentration (C.A.C.), the system transitioned from dispersed, fast-diffusing species to large aggregates with much slower diffusivity.

The DLS analysis data (Figure 4) reveal a clear, Zn(II)-dependent transition: the diffusion coefficient decreases sharply at ~0.5–0.6 μM Zn(II), consistent with the onset of formation of larger species (i.e., Zn(II)-induced self-association/aggregation).

### 2.3. Mass Spectrometry

ESI-MS in positive ion mode was employed to selectively detect Zn(II)–Tβ4 complexes. Prior to analysis, insoluble aggregates were removed by sequential filtration and centrifugation, ensuring that only the soluble fraction of positively charged peptide–metal species entered the electrospray source. Because electrospray ionization preserves non-covalent coordination complexes during ion transfer into the gas phase and enables sensitive detection of low-abundance peptide–metal adducts [43], the method is well-suited for characterizing the stoichiometry and speciation of soluble Zn(II)–Tβ4 complexes.

**Figure 5:**
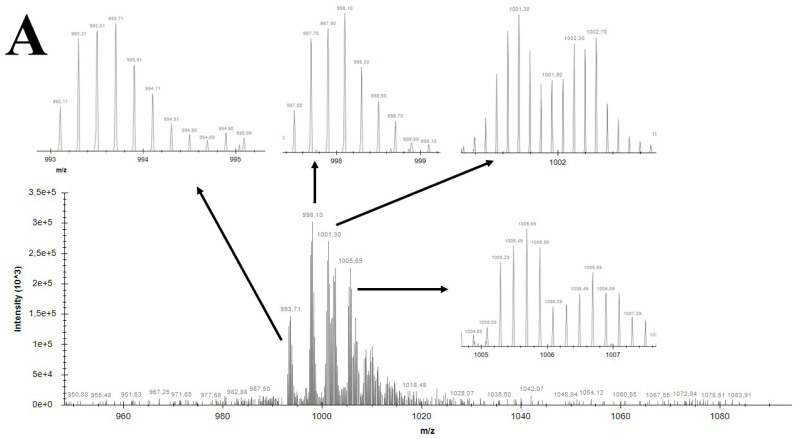

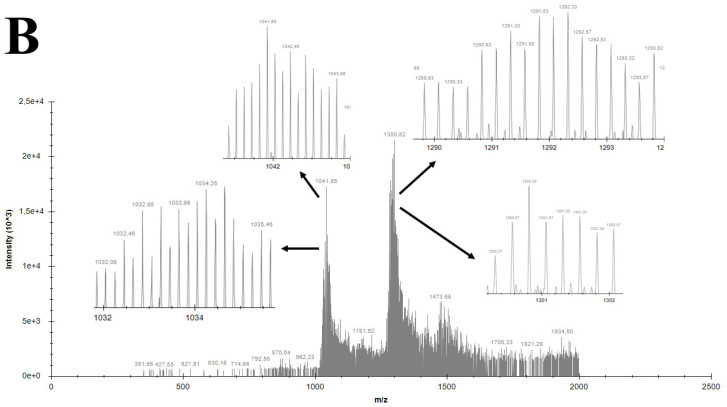
ESI-MS spectrum of free Tβ4 peptide (**A**) and Tβ4 complex with Zn(II) ions (**B**) in positive ion mode.

ESI-MS was used to verify the Zn(II) coordination by Tβ4 at a physiological pH of 7.4. Free Tβ4 spectra (Figure 5A) is of good quality with high signal-to-noise ratios. The molecular formula of Tβ4 in its zero charge form is C_212_H_350_N_56_O_78_S. Tβ4 has 11 carboxylic groups in the side changes in amino acids, 1 carboxylic group at C-end, 9 amine groups of Lys residues and 4 alpha-amine groups of Asp and Glu residues. Thus, Tβ4 is defined as a ligand with 25 labile protons (LH_25_, C_212_H_363_N_56_O_78_S). The ESI-MS spectrum in Figure 5A shows the group of signals at ~993 *m*/*z*, which corresponds to [LH_17_]^5+^ (C_212_H_355_N_56_O_78_S), where 8 out of 25 labile protons are dissociated. Other signals correspond to adducts with sodium ions and water molecules: [LH_16_Na]^5+^ (C_212_H_354_N_56_O_78_SNa), [LH_16_Na(H_2_O)]^5+^ (C_212_H_356_N_56_O_79_SNa), [LH_15_Na_2_]^5+^ (C_212_H_353_N_56_O_78_SNa_2_) and [LH_14_Na_3_]^5+^ (C_212_H_352_N_56_O_78_SNa_3_). The formation of such adducts is a result of high Tβ4 affinity to sodium ions (NaOH and HCl were used to manually fix the pH = 7.4) due to the presence of numerous carboxylic and amine groups in the side chains of amino acids.

The spectrum of Tβ4 with zinc ions (Zn(II) to Tβ4 molar ratio 10:1) in Figure 5B shows a higher signal-to-noise ratio with respect to Figure 5A. This effect is ascribed to the well-known interference of metal cations with the ESI process [44]. Moreover, the intensity of the signals is lower (respect to Figure 5A) due to the solution filtration before the analysis, in order to remove eventual solid precipitates. The signals at ~1041 *m*/*z* Tβ4 of 5+ charge corresponds to the following complexes: [Zn_3_LH_11_]^5+^ (C_212_H_349_N_56_O_78_SZn_3_), [Zn_3_LH_11_(H_2_O)]^5+^ (C_212_H_351_N_56_O_79_SZn_3_), [Zn_3_LH_10_Na]^5+^ (C_212_H_348_N_56_O_78_SZn_3_Na), [Zn_3_LH_9_Na_2_]^5+^ (C_212_H_347_N_56_O_78_SZn_3_Na_2_) and [Zn_3_LH_9_Na_2_(H_2_O)]^5+^ (C_212_H_349_N_56_O_79_SZn_3_Na_2_). Furthermore, ~1300 *m*/*z* present Zn(II) complexes with Tβ4 of 4+ charge and a metal-to-peptide molar ratio of 3:1 of the following formula: [Zn_3_LH_10_(H_2_O)]^4+^ (C_212_H_350_N_56_O_79_SZn_3_), [Zn_3_LH_9_Na]^4+^ (C_212_H_347_N_56_O_78_SZn_3_Na), [Zn_3_LH_9_Na(H_2_O)]^4+^ (C_212_H_349_N_56_O_79_SZn_3_Na), [Zn_3_LH_8_Na_2_]^4+^ (C_212_H_346_N_56_O_78_SZn_3_Na_2_) (Figures S10–S14).

These findings confirm the coordination of three Zn(II) ions per Tβ4 molecule. The presence of multiple adducts with sodium and water molecules further supports the peptide’s polydentate nature and its ability to form stable metal complexes.

It is important to note that the ESI-MS spectra presented here exclusively represent the soluble and ionizable fraction of the Tβ4/Zn(II) system under electrospray conditions. Because aggregated Zn–Tβ4 complexes exhibit extremely low aqueous solubility at physiological pH, the samples were centrifuged prior to MS analysis to remove the insoluble material, which would otherwise obstruct the ESI source and compromise instrument performance. As a consequence, the detected ionic species—including the Zn_3_Tβ4 clusters and their sodium or water adducts—correspond only to the fraction that remained dispersed, solvated, and capable of entering the gas phase as multiply charged ions. The insoluble aggregates characterized by DLS and SEM-EDS are therefore not detected by ESI-MS, which explains the presence of well-resolved Zn-bound complexes despite the concurrent formation of substantial precipitated material in bulk solution.

### 2.4. NMR Spectroscopy

An NMR characterization of Tβ4 was performed using both one- and two-dimensional experiments in the absence and presence of Zn(II) at physiological pH (7.4). We carried out a 1D ^1^H-NMR titration by the incremental addition of Zn(II). As shown in Supplementary Figure S15, no significant chemical shift perturbations were observed upon Zn(II) addition. All resonances remained essentially unchanged, indicating the absence of specific binding-induced structural rearrangements. However, a progressive loss of spectral resolution was detected at higher Zn(II) concentrations, consistent with the onset of metal-induced aggregation, in agreement with DLS results.

**Figure 6:**
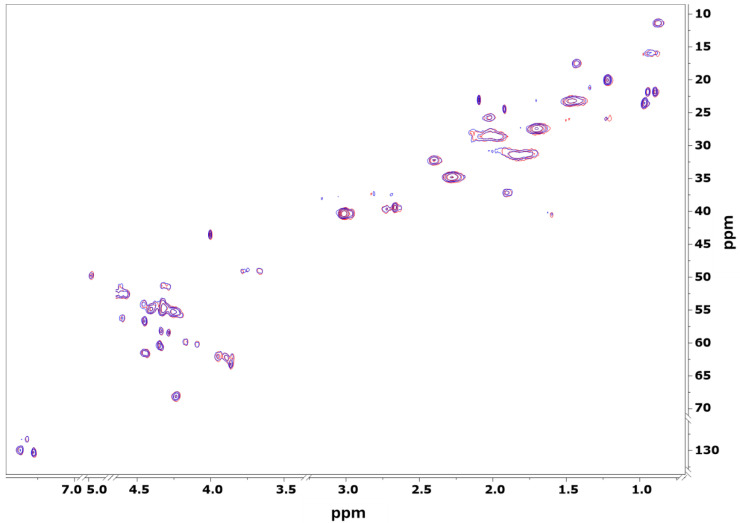
Overlay of the 2D ^13^C–^1^H HSQC spectra for the free form of Tb4 (red) and the sample with Zn(II): Tβ4 at a 1:1 ratio (blue).

Subsequently, two-dimensional ^1^H–^13^C HSQC NMR spectra of Tβ4 (Figure 6) were recorded in the absence and presence of Zn(II) and compared to the literature data [27,40]. The use of D_2_O as solvent was required to reduce background proton signals and improve spectral clarity in the aliphatic region. While this experimental setup precluded observation of backbone amide NH signals, it allowed partial characterization of the soluble fraction of the peptide. Consistent with the 1D experiments, no significant chemical shift perturbations were detected in the HSQC spectra upon Zn(II) addition, indicating that Zn(II) does not induce detectable local or global conformational changes in Tβ4 in solution.

### 2.5. Scanning Electron Microscopy (SEM)

**Figure 7:**
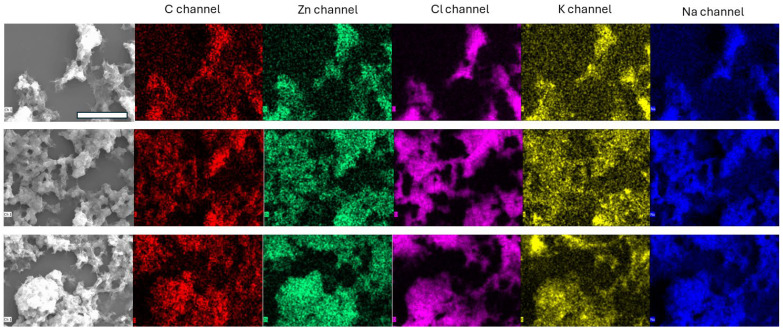
Scanning electron microscopy (SEM) image of Zn(II)/Tβ4 complexes. The scale bar represents 10 µm.

Scanning electron microscopy (SEM) coupled with energy-dispersive X-ray spectroscopy (EDS) was employed to characterize the morphology and elemental composition of the precipitate formed upon Zn(II) binding to Tβ4 (Figure 7). The secondary electron image (SEI) revealed compact, aggregated structures consistent with low-solubility metal–peptide complexes. Elemental mapping confirmed the co-localization of zinc (Zn) with carbon (C), supporting the identification of the precipitate as Zn(II)/Tβ4 complexes.

In addition to Zn and C, the EDS maps showed strong spatial correlation for sodium (Na), potassium (K), and chlorine (Cl), suggesting that these ions are retained within the complex matrix or are associated with the peptide’s ionizable groups. The presence of Na and Cl is consistent with the controlled pH conditions used to maintain physiological pH, while K may reflect residual ionic content from the peptide preparation. The elemental overlap reinforces the interpretation that the observed aggregates are composed of organic material (Tβ4) coordinated with Zn(II) and stabilized by ionic interactions.

These SEM-EDS findings align with the results from DLS and ESI-MS. DLS experiments demonstrated Tβ4/Zn(II) aggregate formation, while ESI-MS confirmed the formation of Zn_3_Tβ4 complexes and revealed multiple charged species with sodium and water adducts. NMR analysis further supported the presence of insoluble aggregates, as the addition of Zn(II) led to the precipitation and loss of spectral resolution.

Together, these data provide converging evidence for the formation of Tβ4/Zn(II) adducts characterized by low solubility and aggregation. SEM-EDS analysis offers direct morphological and compositional validation of the complex formation, complementing the thermodynamic and spectrometric insights.

## 3. Discussion

Thymosin β4 (Tβ4) is an actin-binding peptide known to interact with multiple protein partners; however, its metal-binding properties remain incompletely understood. Previous studies from our group provided an overview of potential metal coordination sites in Tβ4 and investigated its interaction with several metal ions, including Ca(II) [45], Fe(II)/Fe(III), and Al(III) [27]. In those systems, high-field NMR spectroscopy revealed that free Tβ4 lacks a stable secondary or tertiary structure, displaying only limited transient helicity (~20%) and behaving predominantly as an intrinsically disordered peptide. Upon coordination with Fe(II), Fe(III), or Al(III), residue-specific NMR chemical shift perturbations and intensity changes were observed, indicating metal association without inducing folding or structural compaction. No long-range NOEs or stable secondary structure elements emerged, confirming that Tβ4 retained a highly dynamic and conformationally heterogeneous state, even in the presence of strongly Lewis-acidic metal ions. Of note, no precipitation was observed under these conditions.

In the present study, we used zeta potential, DLS, ESI-MS, NMR and SEM to characterize the aggregation process, stoichiometry and morphology of the Zn(II) complexes with Tβ4. In parallel, I-TASSER modeling was used to assess the global foldability of Tβ4. The I-TASSER modeling is fully consistent with both the previously reported NMR data [27] and the present observations. Threading templates showed very low sequence identity and marginal Z-scores, while the final models exhibited low C-scores (−1.24 to −5.0), uniformly high predicted B-factors, and a coil-dominated secondary structure (Supplementary Figures S1 and S2, Tables S1 and S2). These features collectively indicate poor foldability and high intrinsic flexibility, in agreement with the experimentally observed disorder. Furthermore, the lack of model convergence toward a single stable fold in I-TASSER mirrors the ensemble-like behavior observed by NMR and supports the conclusion that Tβ4 does not undergo metal-induced structural compaction.

The combined zeta potential and dynamic light scattering (DLS) analyses provide a coherent physicochemical framework, explaining the aggregation behavior of Tβ4 in the presence of Zn(II) ions at physiological pH. These measurements, interpreted alongside ESI-MS, NMR, and SEM-EDS results, reveal a unified mechanism of metal-driven association. At pH 7.4, free Tβ4 exhibits a markedly negative zeta potential due to the deprotonation of its eleven acidic side chains. The progressive addition of Zn(II) leads to a stepwise reduction in surface charge, reflecting charge neutralization through metal coordination to carboxylate residues.

The DLS measurements quantitatively confirm this transition. Below the C.A.C., Tβ4 exists predominantly as small, monomeric or minimally associated species. As the Tβ4:Zn(II) molar ratio approaches 0.9, DLS reveals an abrupt and substantial increase in hydrodynamic particle size, marking the onset of rapid aggregate formation. Beyond this point, the appearance of broad, multimodal size distributions indicates the development of heterogeneous supramolecular assemblies, consistent with a metal-bridging mechanism rather than monodisperse oligomerization.

ESI-MS provided direct evidence for the fraction of Zn_3_Tβ4 complexes, including multiple polydentate adducts, consistent with carboxylate-based coordination. NMR experiments demonstrated rapid sample precipitation upon Zn(II) addition, preventing further analysis of the backbone amide region—an observation that aligns precisely with the aggregation onset detected by DLS and the electrostatic neutralization seen in zeta potential measurements. Finally, SEM-EDS imaging confirmed that the resulting precipitate consists of compact Zn–Tβ4 aggregates enriched in carbon, oxygen, and zinc, with minimal inorganic contaminants, validating the compositional identity of the aggregated species. The morphology of the precipitate was compacted and irregular in terms of organized structures.

Taken together, these data indicate that Zn(II) does not induce the folding of Tβ4 into a stable tertiary structure; rather, it drives aggregation via charge neutralization and metal-bridging interactions characteristic of intrinsically disordered peptides rich in acidic residues. The convergence of zeta potential, DLS, ESI-MS, NMR, and SEM-EDS findings support a model in which Zn(II) binding serves as a physicochemical switch, transforming Tβ4 from a highly soluble, negatively charged peptide into aggregated Tβ4/Zn(II) complexes once the C.A.C. is reached at physiological pH.

This integrated interpretation highlights a plausible mechanism by which Zn(II) may regulate Tβ4 behavior in biological environments—particularly in extracellular or zinc-rich tissues—through modulation of peptide charge, solubility, and supramolecular organization.

Free zinc (II) ions function as a signaling mediator, leading to the concept that zinc is the “the calcium of the 21st century” [46]. Extracellular zinc increase is a signaling mediator in endocrine, paracrine, and autocrine systems. In pancreatic cells, glucose stimuli lead to zinc ions releasing together with insulin and can further suppress hepatic insulin clearance [47] and reduce insulin secretion from the β-cells [47,48]. In the central nervous system, the excitation of synaptic clefts leads to zinc release from presynaptic neurons, which modulates synaptic transmission by binding to various transporters and receptor channels on postsynaptic neurons [46,49].

**Figure 8:**
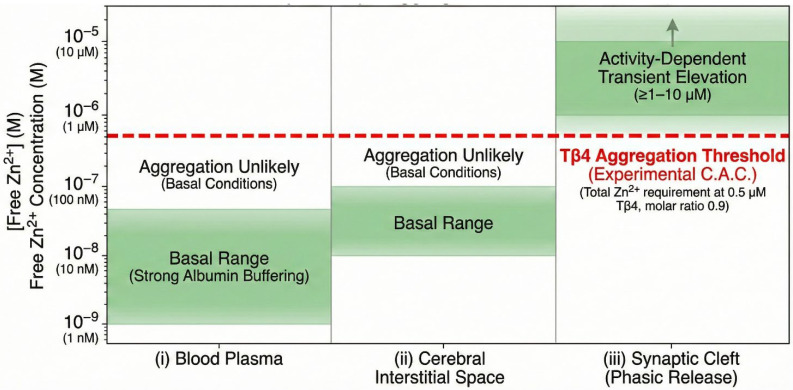
Quantitative relevance map comparing extracellular free Zn^2+^ concentrations with the aggregation threshold (C.A.C.) of Tβ4. This figure presents a quantitative relevance map positioning the experimentally determined critical aggregation concentration (C.A.C.) of Thymosin β4 (Tβ4) relative to physiologically documented ranges of free extracellular Zn(II) in different biological microdomains. Shaded green bands denote the literature-based concentrations of labile Zn(II) in: (i) blood plasma, where free Zn(II) exists in the low-nanomolar range (approximately 1–50 nM) due to strong buffering by albumin; (ii) the brain extracellular (interstitial) space, where basal free Zn(II) levels typically lie between ~10–100 nM; and (iii) synaptic cleft microdomains, in which phasic vesicular release can transiently elevate free Zn^2+^ to ≥1–10 μM during neuronal activity. The red dashed line indicates the C.A.C. for Zn–Tβ4 aggregation at physiological pH, determined experimentally by zeta potential and dynamic light scattering measurements. Under these conditions, aggregation occurs when Tβ4 is 0.5 μM and the Tβ4:Zn(II) molar ratio is 0.9, corresponding to a total Zn(II) requirement of ~0.56 μM for the onset of aggregation. Because Zn(II) is partially bound to Tβ4 and other solution ligands, the free Zn(II) concentration at the C.A.C. is lower than this line; nevertheless, the threshold provides a quantitative benchmark for comparing aggregation propensity to extracellular Zn(II) availability. Overlaying the biological Zn^2+^ ranges with the experimental C.A.C. illustrates that basal extracellular Zn(II) levels in plasma or interstitial brain fluid are substantially below the aggregation threshold, suggesting that Zn-induced Tβ4 aggregation is unlikely under resting physiological conditions. In contrast, synaptic cleft microdomains exhibit transient free Zn(II) elevations that can equal or exceed the Zn(II) availability required for Tβ4 aggregation, indicating that such aggregation events may be favored in highly localized, activity-dependent extracellular environments. This framework provides a quantitative physiological context for interpreting the biophysical behavior of the Zn–Tβ4 system and supports the hypothesis that Tβ4 aggregation may be selectively triggered in Zn-rich microdomains.

To contextualize the aggregation behavior of Tβ4 (Figure 8), we mapped the literature-based ranges of free extracellular Zn(II) against the experimentally determined C.A.C. for Tβ4–Zn(II). Basal brain extracellular Zn(II) is reported in the low-nanomolar range (∼10–100 nM), whereas synaptic cleft transients can reach at least ∼1 μM following activity-dependent release; blood plasma free Zn(II) is maintained in the low-nanomolar regime by albumin buffering [50,51,52].

Our zeta potential and DLS measurements establish that, at physiological pH, the critical aggregation concentration (C.A.C.) is reached when Tβ4:Zn(II) = 0.9 at Tβ4 = 0.5 μM, corresponding to a total Zn requirement of ≈0.56 μM. Because Zn^2+^ partitions among peptide and other ligands, the free Zn(II) at the C.A.C. will be lower than 0.56 μM; nonetheless, the comparison shows that phasic synaptic microdomains (≥1 μM free Zn(II)) can, in principle, exceed the availability threshold for aggregation at sub-micromolar Tβ4, whereas basal interstitial or plasma conditions (nM) are typically below this threshold (Figure 8) [52].

This quantitative framework supports a model in which the Zn(II)-driven aggregation of Tβ4 is unlikely under basal extracellular conditions but becomes plausible in transient synaptic microdomains where free Zn(II) rises into the micromolar range, consistent with our ESI-MS stoichiometry, and zeta/DLS-defined aggregation onset [52].

### 3.1. The Extracellular Zn(II) Landscape in the Brain

Under resting conditions, free (labile) Zn(II) in the brain extracellular space is maintained in the low-nanomolar range by endogenous buffers and transporter networks, consistent with 10–100 nM estimates from in vitro and in vivo studies of hippocampal tissue. In contrast, synaptic release from ZnT3-positive vesicles produces short-lived, spatially confined transients in the cleft that elevate free Zn(II) to ≥1 μM on the millisecond-to-second scale, as inferred from electrophysiological–chelation paradigms and supported by biophysical modeling of the mossy-fiber cleft. Recent genetically encoded and membrane-anchored fluorescent indicators with low-micromolar affinity have further confirmed that micromolar extracellular Zn(II) signals accompany neuronal activity in cortical circuits, underscoring the plausibility of μM-scale Zn(II) microdomains in vivo [52].

### 3.2. Mechanistic Intersections Between Zn(II) Signaling and Tβ4:Zn(II) Aggregation

Extracellular Zn(II) modulates NMDARs, AMPARs, GABA_A, and Gly receptors with bidirectional effects that depend on concentration and receptor subunit context, thereby shaping both excitatory and inhibitory synaptic currents. Micromolar Zn(II) in the cleft can inhibit or potentiate these receptors, and changes to the amplitude or time course of Zn(II) signals alter synaptic integration and plasticity. If Tβ4:Zn(II) assemblies sequester Zn(II) rapidly during a release event, they may blunt the peak Zn(II) signal and attenuate receptor modulation; conversely, slow Zn(II) re-release from aggregates between stimuli could prolong receptor exposure beyond its native window, distorting the temporal coding that Zn(II) provides at synapses [53,54].

Synaptically released Zn(II) also engages the Zn-sensing G-protein-coupled receptor ZnR/GPR39, triggering Ca(II) rises and ERK phosphorylation in hippocampal neurons; these responses are chelator-sensitive and depend on vesicular Zn pools. A perisynaptic Tβ4:Zn(II) phase that captures diffusible Zn(II) could damp ZnR/GPR39 signaling during bursts, whereas Zn(II) liberation from aggregates might prolong or mis-time metabotropic events, with downstream consequences for kinase-dependent plasticity cascades [54].

### 3.3. Consequences for Plasticity and Cognition

The hippocampus, and particularly mossy-fiber–CA3 synapses, is enriched in vesicular Zn and is a canonical locus for Zn-modulated plasticity and memory processes. Reviews and experimental studies converge on the view that synaptic Zn(II) regulates LTP/LTD and is required for aspects of learning and memory, with perturbations in Zn transients leading to cognitive deficits. By inserting a non-native Zn(II) buffer into the perisynaptic space, Tβ4:Zn(II) aggregates could weaken or re-time Zn-dependent gating of receptor and signaling networks, thereby impairing plasticity in Zn-rich circuits such as dentate gyrus–CA3 [53].

### 3.4. Pathophysiological Contexts That Elevate Risk of Crossing the C.A.C.

Several conditions are expected to amplify extracellular Zn(II) transients and hence increase the likelihood of crossing the Tβ4 C.A.C.: (i) Hyperexcitability and seizures increase the frequency and magnitude of Zn(II) release; in vivo imaging and indicator studies show robust synaptic Zn(II) signals under such conditions, placing microdomains well within the μM regime [55]; (ii) aging is accompanied by changes in extracellular Zn homeostasis; reviews link altered Zn dynamics to age-related cognitive decline, suggesting a narrowed safety margin between basal Zn(II) and the aggregation threshold in aged hippocampus [56].

These contexts reinforce the specific vulnerability of brain synapses to Zn(II)-driven Tβ4 aggregation, in contrast to blood plasma, where albumin constrains free Zn(II) to the low-nanomolar range, well below the C.A.C. [57].

### 3.5. Biophysical Effects in the Perisynaptic Microenvironment

Beyond Zn(II) chemistry, proteinaceous assemblies at the perisynaptic/extracellular-matrix interface can alter diffusion geometries and ligand access. The modeling of cleft Zn(II) shows that the spatiotemporal profile of a Zn burst depends sensitively on binding partners and geometry; introducing an immobile aggregate phase is predicted to extend Zn residence times locally and to reshape the concentration–time waveform experienced by receptors and transporters. Such effects could synergize with the Zn-binding capacity of Tβ4:Zn(II) aggregates to produce nonlinear distortions of Zn signaling during trains of activity [58].

### 3.6. Potential for Maladaptive Plasticity and Neurotoxicity

While low-nanomolar extracellular Zn(II) supports normal synaptic function, sustained micromolar exposure or mis-timed Zn transients have been associated with receptor dysregulation, intracellular Zn loading, and neurotoxic cascades under pathological conditions. By capturing Zn(II) during spikes and releasing it outside the physiological window, Tβ4:Zn(II) aggregates may tilt the balance toward maladaptive plasticity and signaling impairment, providing a potential mechanistic link between Zn dyshomeostasis and cognitive decline in vulnerable circuits.

**Figure 9:**
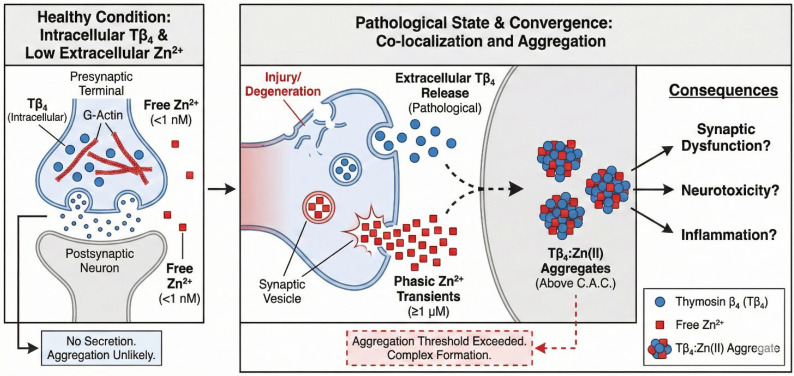
Schematic representation of the conditions enabling extracellular Thymosin β4 (Tβ4) to encounter micromolar Zn(II) transients in the synaptic cleft. Under physiological conditions, Tβ4 is an intracellular G-actin-sequestering peptide not released through synaptic vesicle exocytosis. However, pathological processes, including neurodegeneration (e.g., APP/PS1 and AD models), reactive gliosis, hyperexcitability and seizure activity, or traumatic brain injury/ischemia, can lead to cell membrane disruption, neuritic degeneration, or glial activation, resulting in elevated extracellular Tβ4 levels in perisynaptic regions. In parallel, Zn-rich synapses generate activity-dependent Zn(II) microdomains, reaching ≥1 μM free Zn(II) during synaptic release, as demonstrated in electrophysiological–chelation assays, engineered synapse models, and quantitative synaptic Zn modeling. When extracellular Tβ4 becomes available in such Zn-rich clefts and total Zn(II) surpasses the experimentally determined critical aggregation concentration (C.A.C.) for Tβ4:Zn(II) complex formation, Tβ4:Zn(II) aggregates may form within the synaptic cleft. The illustration highlights the convergence of pathological Tβ4 release and physiological micromolar Zn(II) transients, providing a mechanistic basis for the localized formation of metal–peptide aggregates in diseased neural tissue.

Collectively, the literature on extracellular Zn(II) dynamics indicates that synaptic microdomains in the brain can reach ≥1 μM free Zn(II) during activity, a regime that overlaps with the Zn availability required to trigger Tβ4:Zn(II) aggregation. In such microdomains (Figure 9), aggregate nucleation would be expected to buffer, re-time, and spatially distort Zn(II) signals, thereby perturbing both ionotropic (NMDAR/AMPAR/GABA_A/GlyR) and metabotropic (ZnR/GPR39) pathways central to plasticity and cognition. The same framework predicts minimal risk of aggregation in compartments where free Zn(II) remains nanomolar (e.g., plasma; interstitial basal brain), but heightened risk during hyperexcitability or aging, when Zn(II) transients are amplified. These considerations provide a mechanistic and quantitative rationale for focusing future work on brain synapses as the primary in vivo context for Tβ4:Zn(II) aggregation and its functional consequences [54,55,58,59].

### 3.7. Potential Situations for Presynaptic Exposure to Tβ4 and the Likelihood of Zn(II)-Driven Aggregation

Tβ4 is not a classical synaptic secretory peptide. Mechanistically, Tβ4 is a cytosolic, highly conserved G-actin-sequestering protein. Its canonical localization is intracellular (cytoplasm and, in some contexts, nucleus), and there is no evidence for activity-dependent exocytosis into the synaptic cleft under normal physiology. Accordingly, any elevation in extracellular/perisynaptic Tβ4 is most plausibly secondary to cell stress, injury, or degeneration, rather than to a dedicated release pathway [60].

### 3.8. Pathological Contexts That Could Elevate Tβ4 at or near Presynaptic Sites

In transgenic mouse models of AD (APP/PS1), brain Tβ4 protein levels are elevated, and glial activation/phenotypic shifts are prominent, consistent with disease-associated remodeling of intracellular peptide pools and potential leakage/redistribution to extracellular compartments. In complementary organoid and mouse studies, TMSB4X/Tβ4 expression is dynamically altered and exogenous Tβ4 can modulate disease phenotypes—further supporting a tight link between Tβ4 biology and degenerative stress. In such settings, synaptic dystrophy and membrane compromise provide a route for cytosolic Tβ4 to access perisynaptic/cleft regions, where micromolar Zn^2+^ transients are known to occur [27].

Activity bursts increase both the frequency and amplitude of synaptic Zn(II) transients, documented in brain slices and in vivo with engineered and genetically encoded indicators tuned to the low-micromolar range. Hyperexcitable tissue is also more vulnerable to structural and metabolic stress that can compromise membranes, increasing the likelihood of intracellular peptide egress into the extracellular space. These conditions jointly elevate the probability that Tβ4 encounters μM Zn(II) microdomains at synapses [61].

TBI/ischemia are characterized by acute membrane disruption, cytosolic leakage, and glial activation, and Tβ4 is repeatedly implicated in CNS repair/regeneration following such insults. These pathologies pragmatically provide (i) a source of extracellular Tβ4 from damaged neurons/glia and (ii) aberrant Zn dynamics associated with excitotoxic signaling—two prerequisites for local supersaturation with respect to the Tβ4 C.A.C.

## 4. Material and Methods

### 4.1. Reagents

All reagents (NaOD, DCl, ZnCl_2_, buffer solutions) were purchased from Sigma Aldrich (St. Louis, MO, USA). Thymosin beta 4 (Thymosin β4 acetate (75591-33-4 free base) was purchased from Prodotti Gianni (last access on: 27 February 2025; https://ricerca.prodottigianni.com/life-science-index.php).

### 4.2. I-TASSER

The I-TASSER server (http://zhanglab.ccmb.med.umich.edu/I-TASSER; last entry on 19 February 2025) [62] is an online platform created for automated protein structure prediction and structure-based function annotation. I-TASSER recognizes structural templates from the Protein Database (PDB; https://www.rcsb.org/, last access on: 28 February 2025) by the use of multiple threading alignments. Full-length structure models are successively built by fragment assembly simulations. The functional insights are finally derived by matching the predicted structure models with known proteins in the function databases. Additionally, I-TASSER predicts ligand-binging sites by COFACTOR [63] that extrapolate binding sites from homologous templates detected by global and local structure comparisons [64].

### 4.3. Dynamic Light Scattering (DLS)

Dynamic light scattering (DLS) measurements were performed using a Malvern Zetasizer Nano (Malvern Instruments Ltd., Worcestershire, UK) operating in backscattering configuration (173°) at 25 °C (instrument temperature control). Tβ4 peptide was prepared in ultrapure water under salt-free, unbuffered conditions at a final concentration of 0.5 µM. A ZnCl_2_ stock solution was prepared in ultrapure water and diluted immediately before use. Zn(II) was titrated stepwise into the peptide solution to obtain final Zn(II) concentrations spanning 0–1.5 µM. After each addition, samples were gently mixed and allowed to equilibrate for [t] min at 25 °C prior to measurement.

For each Zn(II) concentration, 5–7 independent measurements (replicates) were acquired, and the translational diffusion coefficient (D) was obtained by cumulant analysis of the intensity autocorrelation functions using the instrument software. Results are reported as mean ± SD. The abrupt decrease in D observed at ~0.5–0.6 µM Zn(II) was used to define the critical aggregation concentration (C.A.C.).

### 4.4. Zeta Potential

Zeta potential measurements were carried out on a Malvern Zetasizer Nano (Malvern Instruments Ltd., Worcestershire, UK) by electrophoretic light scattering using disposable folded capillary cells at 25 °C. Tβ4 peptide solutions were prepared in ultrapure water without added buffer or salt at a final concentration of 0.5 µM. ZnCl_2_ was added stepwise to reach final Zn(II) concentrations between 0 and 1.5 µM. The pH was monitored after each Zn(II) addition using a microelectrode and remained within 7.48–7.74 throughout the titration. Electrophoretic mobility values were converted to zeta potential (ζ) using the Hückel approximation as implemented in the instrument software. For each Zn(II) concentration, 5–7 replicate measurements were performed and data are reported as mean ± SD.

### 4.5. Mass Spectrometry

The qualitative investigation of Tβ4 and its complexes with Zn(II) and Cu(II) ions was performed by an ion mobility MS system. Positive ESI-MS full scan spectra were recorded on a high-resolution LTQ Orbitrap Elite^TM^ mass spectrometer (Thermo Fisher Scientific, Waltham, MA, USA). The solutions were infused into the ESI source at a flow rate of 5.00 μL/min. Spectra were recorded with a resolution of 240,000 (FWHM). Instrument conditions were as follows: spray voltage 5000 V, capillary temperature 275 °C, sheath gas 10 (arbitrary units), auxiliary gas 5 (arbitrary units), sweep gas 0 (arbitrary units), probe heater temperature 45 °C. Precursor ions of peptide and complex were selected and fragmentated at different collision induced dissociation (CID) energies in MS/MS experiments.

All samples were prepared in pure water at physiological pH = 7.4. The pH measure was executed with the daily calibrated Methrom electrode. The pH was fixed manually by the addition of NaOH and/or HCl. The final Tβ4 concentration was 0.1 mM. The zinc-complex formation study was made with the solution containing ZnCl_2_ and Tβ4 in the molar ratio 10:1.

Mass spectra simulations were performed with open-source platform Envipath and are presented in Supplementary Materials (Figures S1–S12).

### 4.6. Nuclear Magnetic Resonance (NMR)

Tβ4 was dissolved in 1200 µL of D_2_O to obtain a final 0.66 mM concentration. The pH of the peptide solution was manually adjusted to 7.51 by the addition of NaOD and/or DCl, as measured using a daily calibrated Metrohm pH-meter (Metrohm AG, Herisau, Switzerland). Successively, the solution was divided into two aliquots of 600 µL each. One aliquot was used to acquire NMR spectra of the free peptide, while the second aliquot was used to prepare a concentrated ZnCl_2_ solution. The zinc solution was then added stepwise to the Tβ4 sample to perform the titration at increasing metal-to-peptide molar ratios. The final Zn(II) concentration ranged from 0 to 3.86 mM.

^1^H NMR spectra were recorded on a Bruker AVANCE III HD 600 spectrometer at 300 K. One-dimensional NOESY presat experiments utilized an acquisition time of 2 s, a relaxation delay of 4 s, and 256 transients. An external standard was prepared by adding 5 µL of a 10 mM aqueous solution of 3-(trimethylsilyl)propionic-2,2,3,3-d_4_ acid sodium salt (100% D_2_O). Heteronuclear ^1^H-^13^C HSQC experiments were conducted with a mixing time of 16 ms, acquiring 2048 and 360 data points in the proton and carbon dimensions, respectively, with a recycle delay of 1 s. Data were apodized using a sine function and zero-filled to 4096 points in the proton dimension and 1024 points in the carbon dimension.

### 4.7. Scanning Electron Microscopy (SEM)

The Zn(II)/Tβ4 precipitate resulted in NMR analysis, and was filtered and dried on glass slides in a Vacuum Oven (ISCO NSV9000, Teledyne ISCO, Lincoln, NE, USA) for 2 days at room temperature in order to reduce crystalline reworking as much as possible. Then the slides were mounted on aluminum support (stub) with double-sided tape. The morphology and elemental composition of the precipitate was analyzed with a scanning electron microscope (SEM) (Sigma 300, Zeiss, Oberkochen, Germany) in combination with energy-dispersive X-ray spectrometry (EDX XFlash detector 630 M, Bruker Nano Gmbh, Berlin, Germany).

## 5. Conclusions

The present study provides the first experimental demonstration that Thymosin β4 (Tβ4), an intrinsically disordered and strongly acidic peptide, can coordinate Zn(II) and form well-defined complexes that subsequently aggregate once a critical aggregation concentration (C.A.C.) is exceeded. Although earlier hypotheses suggested that Tβ4 may interact with metal ions based on its sequence composition and indirect biochemical observations, no prior work had experimentally validated the existence of discrete Tβ4/Zn(II) complexes or shown that these assemblies undergo metal-driven aggregation. Using a combination of zeta potential, dynamic light scattering, electrospray ionization mass spectrometry, NMR analyses, and SEM/EDS imaging, we demonstrate that Zn(II) progressively neutralizes the negative surface charge carried by the peptide’s abundant carboxylate residues. This process culminates in a peptide-to-metal ratio of approximately 0.9, precisely where DLS reveals a sharp decrease in diffusion coefficient and SEM confirms the formation of compact, low-solubility aggregates consistent with metal-bridged peptide networks.

ESI-MS shows that Tβ4 forms with zinc ion complexes with a clear 3:1 stoichiometry and multiple polydentate binding interactions, supporting the conclusion that the peptide coordinates Zn(II) primarily via its Asp and Glu. NMR experiments further confirm that Zn(II) binding does not induce folding or structured conformational organization; rather, the disappearance of resonances reflects aggregation of the Zn-bound population. Together, these findings establish a physicochemical framework in which Zn(II) acts as a coordination bridge and charge-neutralizing agent, transforming soluble monomeric Tβ_4_ into aggregated assemblies when Zn availability surpasses a well-defined threshold.

By comparing this C.A.C. with physiologically documented extracellular Zn(II) concentrations, we show that Zn-induced aggregation is unlikely to occur in basal plasma or interstitial fluids, where free Zn(II) remains in the nanomolar range. However, synaptic microdomains in the brain experience transient bursts of free Zn(II), reaching or exceeding 1 μM during neuronal activity—well above the Zn requirement for aggregation. When considered alongside pathological events that elevate extracellular Tβ4 levels (including neurodegeneration, seizures, or traumatic injury), our findings introduce the novel hypothesis that Tβ4:Zn(II) aggregation may occur in vivo within Zn-rich synaptic niches. This possibility expands the biological significance of Tβ4 and raises the prospect that Zn-mediated supramolecular assembly could influence extracellular metal buffering, synaptic signaling, or neuroinflammatory processes in disease contexts. Ultimately, this study contributes a fundamentally new dimension to Tβ4 biology by identifying metal-induced aggregation as a previously unrecognized behavior of this peptide.

## 6. Future Directions

Moving forward, several complementary lines of investigation are needed to determine whether the aggregation phenomena characterized here are relevant to living systems and whether they contribute to physiological or pathological pathways. A central priority will be to define the kinetic parameters governing Zn(II) binding, aggregate nucleation, and Zn release. Understanding the temporal behavior of these processes—particularly whether Tβ4/Zn assemblies act as fast Zn buffers or slow Zn reservoirs—will help clarify their potential to influence Zn fluxes in the synaptic cleft, where the biological lifetime of Zn signals spans milliseconds to seconds. Quantitative kinetic analyses using time-resolved spectroscopies and competitive ligand displacement will provide critical insights into the mechanistic interplay between Zn availability and aggregate stability.

Parallel efforts will be required in cellular and organotypic models, where exposure of neurons, astrocytes, and microglia to Tβ4–Zn(II) mixtures may reveal biological consequences of aggregation, including effects on viability, inflammation, Zn homeostasis, or receptor modulation. Acute hippocampal slices, equipped with modern fluorescent Zn indicators, offer a system in which the impact of exogenous Tβ4 on endogenous Zn transients can be directly assessed. Electrophysiological studies may further determine whether Tβ4 aggregates perturb the Zn-dependent modulation of NMDA, AMPA, GABA_A, or ZnR/GPR39 pathways, thereby influencing synaptic plasticity.

To assess physiological relevance more broadly, it will be essential to examine pathological in vivo models. Neurodegenerative diseases such as Alzheimer’s disease, experimental seizure paradigms, and traumatic brain injury or ischemia all feature synaptic stress, Zn dyshomeostasis, and the potential release of intracellular peptides into extracellular space. These conditions may permit extracellular Tβ_4_ to cross the aggregation threshold in the presence of synaptically released Zn. Quantifying Tβ4 abundance in extracellular fractions and determining whether its levels correlate with Zn-rich regions or synaptic degeneration will help bridge the gap between in vitro findings and in vivo mechanisms.

Finally, post mortem human brain studies represent a crucial direction for validating the in vivo relevance of Tβ4/Zn(II) aggregation. Immunohistochemical analyses targeting Tβ4, when combined with Zn-specific staining methods and synaptic or neuropathological markers, could reveal whether Tβ4 aggregates accumulate in Zn-rich brain regions affected by neurodegenerative diseases. Proteomic profiling of insoluble fractions may detect endogenous Tβ4-containing assemblies, providing direct evidence for or against the occurrence of the aggregation mechanism identified in vitro.

Together, these future studies will establish whether the Tβ4/Zn(II) aggregation pathway uncovered here is simply a physicochemical curiosity or a biologically meaningful process contributing to synaptic dysfunction, neuroinflammation, or broader metal–protein interactions in the human brain.

## Data Availability

The original contributions presented in this study are included in the article/Supplementary Materials. Further inquiries can be directed to the corresponding author.

## Supplementary Materials

The following supporting information can be downloaded at: https://www.mdpi.com/article/10.3390/ijms27041740/s1.
