# Supplementary material for: Zinc Coordination by Thymosin β4: Structural Determinants and Functional Implications
Source: Int J Mol Sci. 2026 Feb 11;27(4):1740. doi: 10.3390/ijms27041740 (PMC12940511; doi:10.3390/ijms27041740)
Supplement: Supplementary file 1 [file ijms-27-01740-s001.zip › ijms-4007830-supplementary.pdf]

Zinc Coordination by Thymosin β4: Structural Determinants and Functional Implications.

Joanna Izabela Lachowicz[*,1], Terenzio Congiu[2], Andrea Salis[3], Flaminia Cesare-Marincola[3]

[1]*Department of Environmental Health, Occupational Medicine and Epidemiology, Wroclaw Medical University, Mikulicza-Radeckiego 7, Wroclaw, PL 50-368, Poland*

[2]*Department of Medical Science and Public Health, University of Cagliari, Cittadella Universitaria, 09042 Monserrato (CA), Italy*

[3]*Department of Chemical and Geological Sciences, University of Cagliari, Cittadella Universitaria, 09042 Monserrato (CA), Italy*

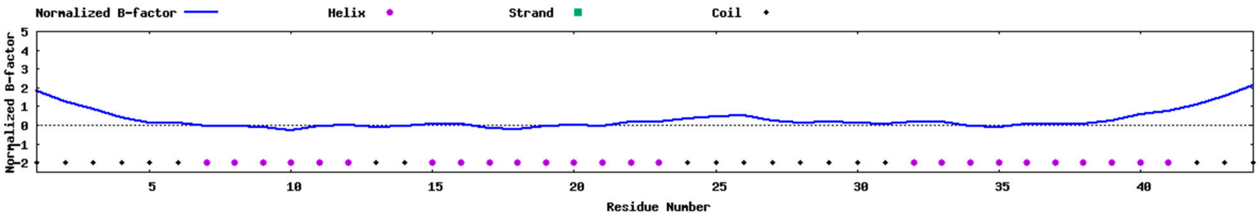

Figure S1. B-factor is a value to indicate the extent of the inherent thermal mobility of residues/atoms in proteins. In I-TASSER, this value is deduced from threading template proteins from the PDB in combination with the sequence profiles derived from sequence databases. The reported B-factor profile in the figure below corresponds to the normalized B-factor of the target protein, defined by B=(B'-u)/s, where B' is the raw B-factor value, u and s are respectively the mean and standard deviation of the raw B-factors along the sequence.

Table S1. I-TASSER modeling starts from the structure templates identified by LOMETS from the PDB library. LOMETS is a meta-server threading approach containing multiple threading programs, where each threading program can generate tens of thousands of template alignments. I-TASSER only uses the templates of the highest significance in the threading alignments, the significance of which are measured by the Z-score, i.e. the difference between the raw and average scores in the unit of standard deviation. The templates in this section are the 10 best templates selected from the LOMETS threading programs. Usually, one template of the highest Z-score is selected from each threading program, where the threading programs are sorted by the average performance in the large-scale benchmark test experiments

Rank	PDB Hit	Iden1	Iden2	Cov	Norm. Z-score	20 | 40 |
Sec.Str<br>Seq						CCCCCCHHHHHHCCHHHHHHHHHCCCCCCCCHHHHHHHHHHCCC<br>MSDKPDMAEIEKFDKSKLKKTETQEKNPLPSKETIEQEKQAGES
1	2kwyA	0.19	0.20	0.98	1.11	MDNAGGVGELEKKAEAGVQGELAEIKK-IAEKKKDDVVKILIET
2	1hj0A	0.78	0.73	0.93	1.31	-ADKPDLGEINSFDKAKLKKTETQEKNTLPTKETIEQEKQAK--
3	1hj0A	0.78	0.73	0.93	3.04	-ADKPDLGEINSFDKAKLKKTETQEKNTLPTKETIEQEKQAK--
4	5l4gK	0.18	0.23	1.00	1.11	LSFLGPEPEDLEDLYSRYKKLQQELEFLEVQEEYIKDEQKNLKK
5	1hj0A	0.78	0.73	0.93	1.29	-ADKPDLGEINSFDKAKLKKTETQEKNTLPTKETIEQEKQAK--
6	2l5gB	0.17	0.16	0.93	1.09	LSKE---ELIQNMDRVDREITMVEQQISKLKKKQQQLEEEAAKP
7	4gdoA	0.12	0.23	0.95	1.09	--SHMRAEERERLAEVEAALEKQRQLAEAHAQAKAQAEREAKEL
8	4pl7A	0.90	0.89	0.93	1.21	EYDESDMAEIEKFDKSKLKKTETQEKNPLPSKETIEQEKQA---
9	4n3xA	0.16	0.16	0.98	1.06	MYKNLDLSQLNERQERIMNEAKKLEKDLIDWTDGIAREVQDIV-
10	5xtcQ	0.11	0.16	1.00	1.01	RQWQPDVEWAQQFGGAVMYPSKETAHWKPPPWNDVDPPKDTIVK

Table S2. For each target, I-TASSER simulations generate a large ensemble of structural conformations, called decoys. To select the final models, I-TASSER uses the SPICKER program to cluster all the decoys based on the pair-wise structure similarity, and reports up to five models which corresponds to the five largest structure clusters. The confidence of each model is quantitatively measured by C-score that is calculated based on the significance of threading template alignments and the convergence parameters of the structure assembly simulations. C-score is typically in the range of [-5, 2], where a C-score of a higher value signifies a model with a higher confidence and vice-versa. TM-score and RMSD are estimated based on C-score and protein length following the correlation observed between these qualities. Since the top 5 models are ranked by the cluster size, it is possible that the lower-rank models have a higher C-score in rare cases. Although the first model has a better quality in most cases, it is also possible that the lower-rank models have a better quality than the higher-rank models as seen in our benchmark tests. If the I-TASSER simulations converge, it is possible to have less than 5 clusters generated; this is usually an indication that the models have a good quality because of the converged simulations

**Model 1**	**Model 2**	**Model 3**	**Model 4**	**Model 5**
C-score=-1.24<br>stimated TM-score = 0.56±0.15<br>Estimated RMSD = 4.8±3.1Å	C-score = -2.37	C-score = -1.53	C-score = -1.58	C-score = -5

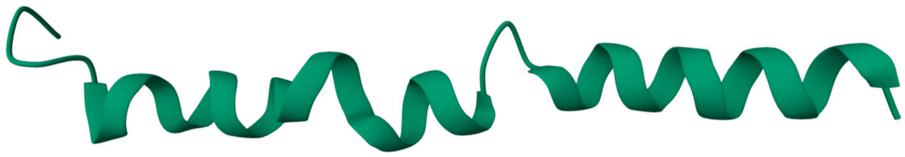

Model 1

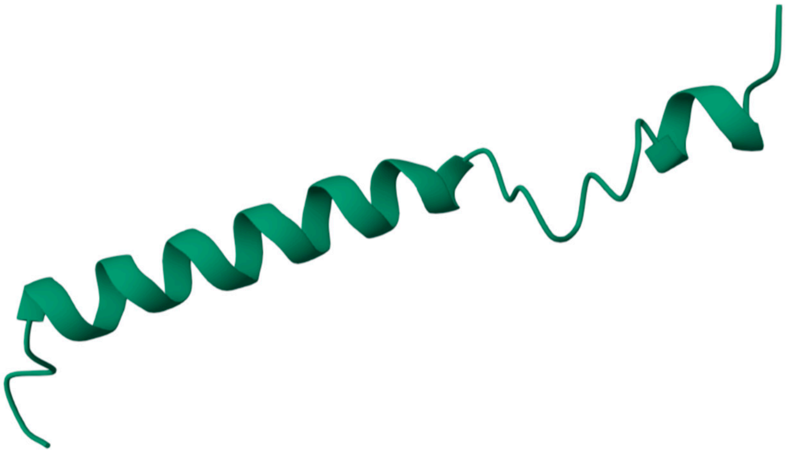

Model 2

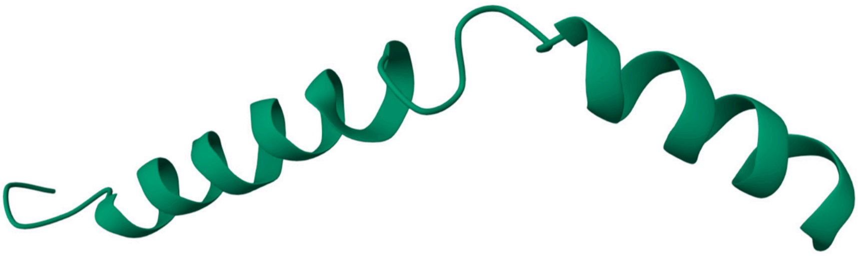

Model 3

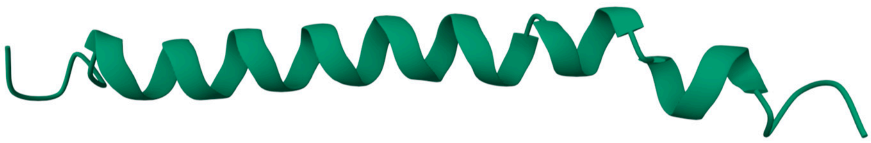

Model 4

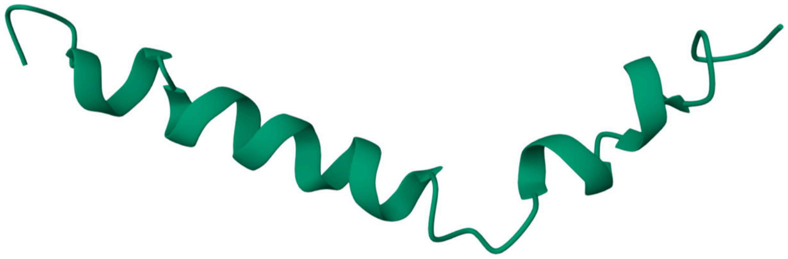

Model 5

Figure S2. Graphical representation of five I-TASSER predicted models.

Table S3. After the structure assembly simulation, I-TASSER uses the TM-align structural alignment program to match the first I-TASSER model to all structures in the PDB library. This section reports the top 10 proteins from the PDB that have the closest structural similarity, i.e. the highest TM-score, to the predicted I-TASSER model. Due to the structural similarity, these proteins often have similar function to the target. However, users are encouraged to use the data in the next section 'Predicted function using COACH' to infer the function of the target protein, since COACH has been extensively trained to derive biological functions from multi-source of sequence and structure features which has on average a higher accuracy than the function annotations derived only from the global structure comparison

Rank	PDB Hit	TM-score	RMSD[a]	IDEN[a]	Cov
1	5y05A	0.637	2.47	0.047	0.977
2	4jleA2	0.629	2.39	0.093	0.977
3	6jfkA	0.623	2.50	0.047	0.977
4	5aj3d	0.622	2.37	0.091	0.977
5	5oqlB	0.620	2.30	0.048	0.955
6	5bqnA2	0.616	2.42	0.163	0.977
7	5twvB	0.609	2.55	0.045	1.000
8	3pltA	0.607	2.55	0.023	0.977
9	5lefC	0.606	2.55	0.093	0.977
10	2xhlB	0.605	2.51	0.070	0.977

Table S4. This section reports biological annotations of the target protein by COFACTOR and COACH based on the I-TASSER structure prediction. While COFACTOR deduces protein functions (ligand-binding sites, EC and GO) using structure comparison and protein-protein networks, COACH is a meta-server approach that combines multiple function annotation results (on ligand-binding sites) from the COFACTOR, TM-SITE and S-SITE programs. (a) C-score is the confidence score of the prediction. C-score ranges [0-1], where a higher score indicates a more reliable prediction. (b) Cluster size is the total number of templates in a cluster. (c)Lig Name is name of possible binding ligand. Click the name to view its information in the BioLiP database. (d) Rep is a single complex structure with the most representative ligand in the cluster, i.e., the one listed in the Lig Name column.

Mult is the complex structures with all potential binding ligands in the cluster.

Rank	C-score	Cluster size	PDB Hit	Lig Name	Ligand Binding Site Residues
1	0.19	14	3kwoA	ZN	21,25
2	0.08	6	4ncfA	MG	20,21
3	0.07	5	4evdA	CD	10,11,14
4	0.07	5	2x2vA	DPV	20,24
5	0.04	3	4auwB	Nuc.Acid	20,23,24,27,29,32,36,39,40

(a) $Cscore^{EC}$ is the confidence score for the EC number prediction. $Cscore^{EC}$ values range in between [0-1]; where a higher score indicates a more reliable EC number prediction.
(b) TM-score is a measure of global structural similarity between query and template protein.
(c) $RMSD^a$ is the RMSD between residues that are structurally aligned by TM-align.
(d) $IDEN^a$ is the percentage sequence identity in the structurally aligned region.
(e) Cov represents the coverage of global structural alignment and is equal to the number of structurally aligned residues divided
by length of the query protein.

Rank	$Cscore^{EC}$	PDB Hit	TM-score	$RMSD^a$	$IDEN^a$	Cov	EC Number
1	0.195	2np0A	0.601	2.54	0.048	0.955	3.4.24.69
2	0.192	3dy5A	0.560	2.84	0.093	0.977	1.13.11.40 4.2.1.92
3	0.191	2uxwA	0.564	2.61	0.024	0.955	1.3.99.-
4	0.186	1n40A	0.562	2.45	0.093	0.864	1.14.-.-
5	0.177	3e6iA	0.566	2.78	0.000	0.932	1.14.13.-

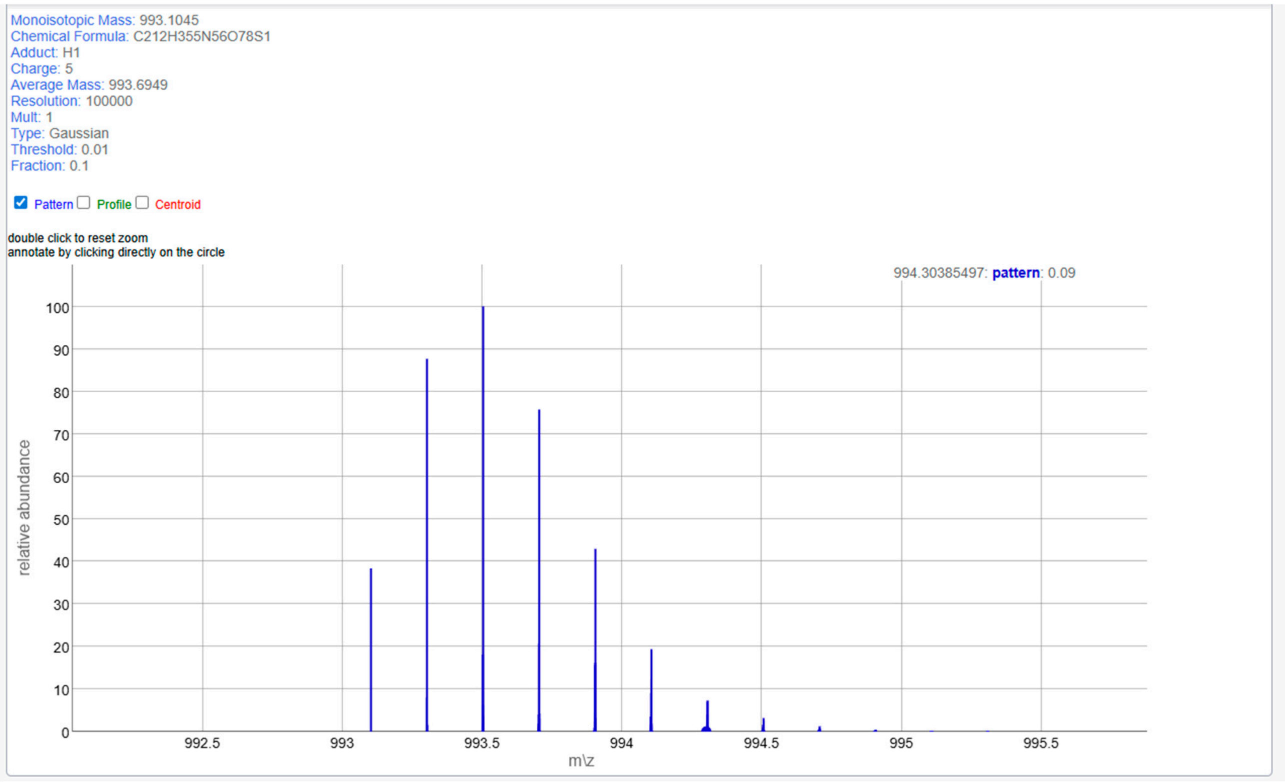

Figure S3. Mass spectra simulation of $[LH_{17}]^{5+}$ (C212H355N56O78S) ion. The simulation was prepared with open source platform Envipat. https://www.envipat.eawag.ch/index.php (last access on 2025-11-05).

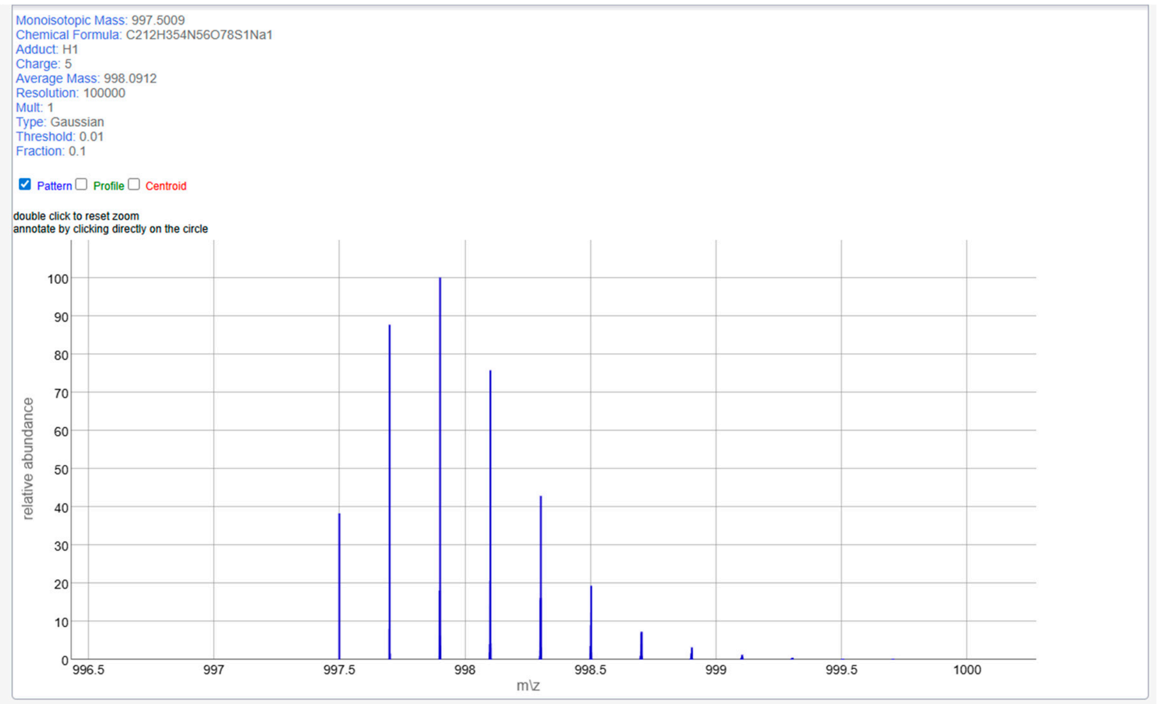

Figure S4. Mass spectra simulation of $[LH_{16}Na]^{5+}$ (C212H354N56O78SNa) ion. The simulation was prepared with open source platform Envipat. https://www.envipat.eawag.ch/index.php (last access on 2025-11-05).

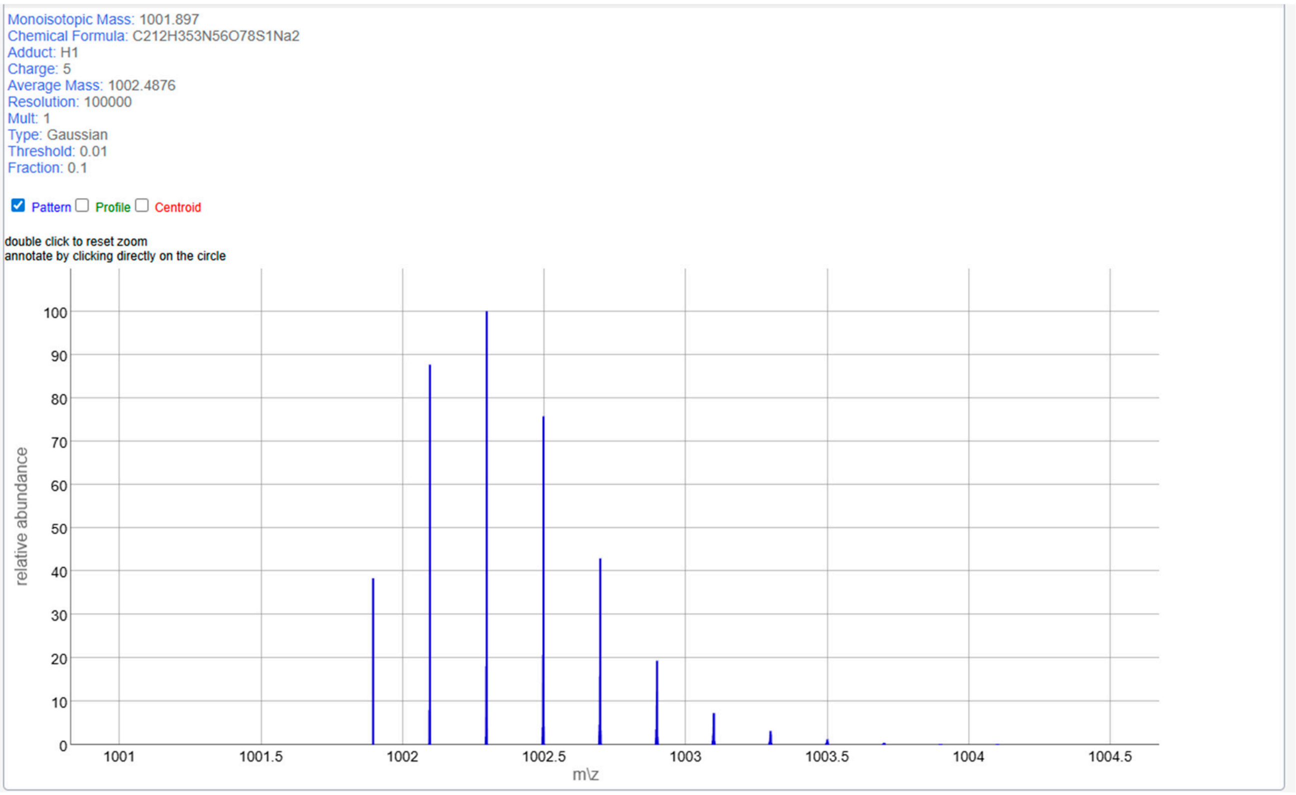

Figure S5. Mass spectra simulation of $[LH_{15}Na_2]^{5+}$ (C212H353N56O78SNa2) ion. The simulation was prepared with open source platform Envipat. https://www.envipat.eawag.ch/index.php (last access on 2025-11-05).

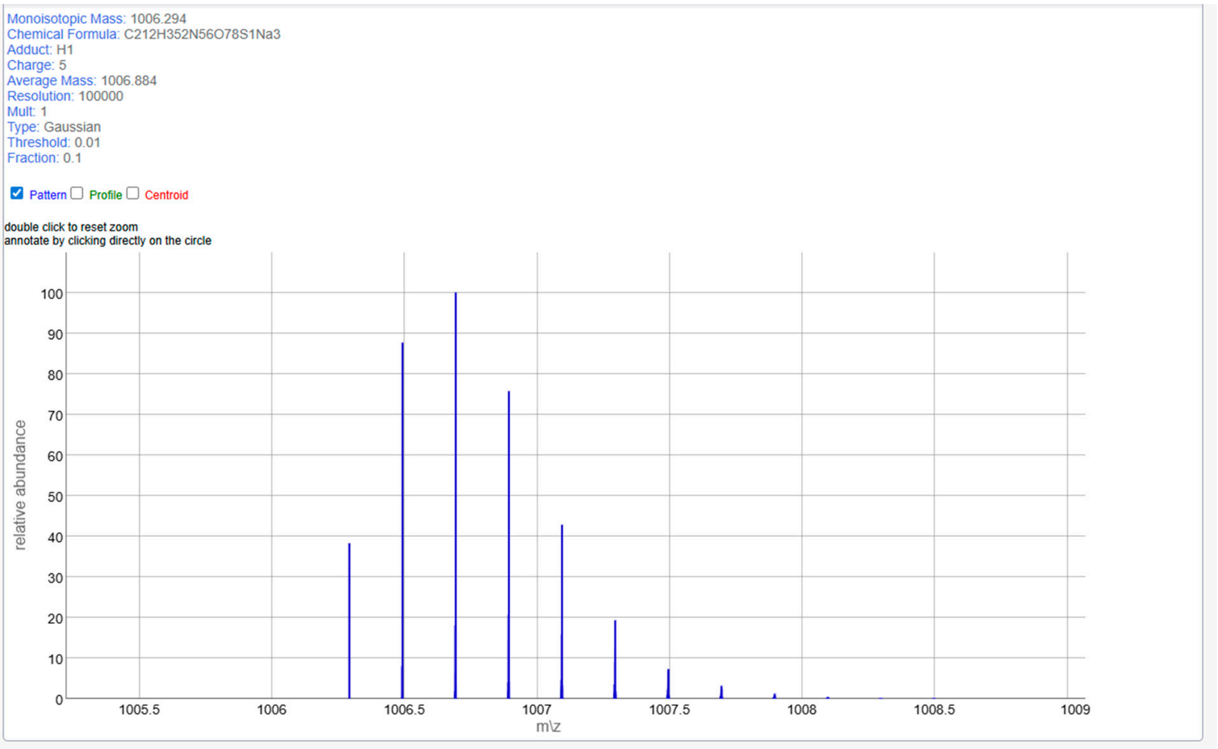

Figure S6. Mass spectra simulation of $[LH_{14}Na_3]^{5+}$ (C212H352N56O78SNa3) ion. The simulation was prepared with open source platform Envipat. https://www.envipat.eawag.ch/index.php (last access on 2025-11-05).

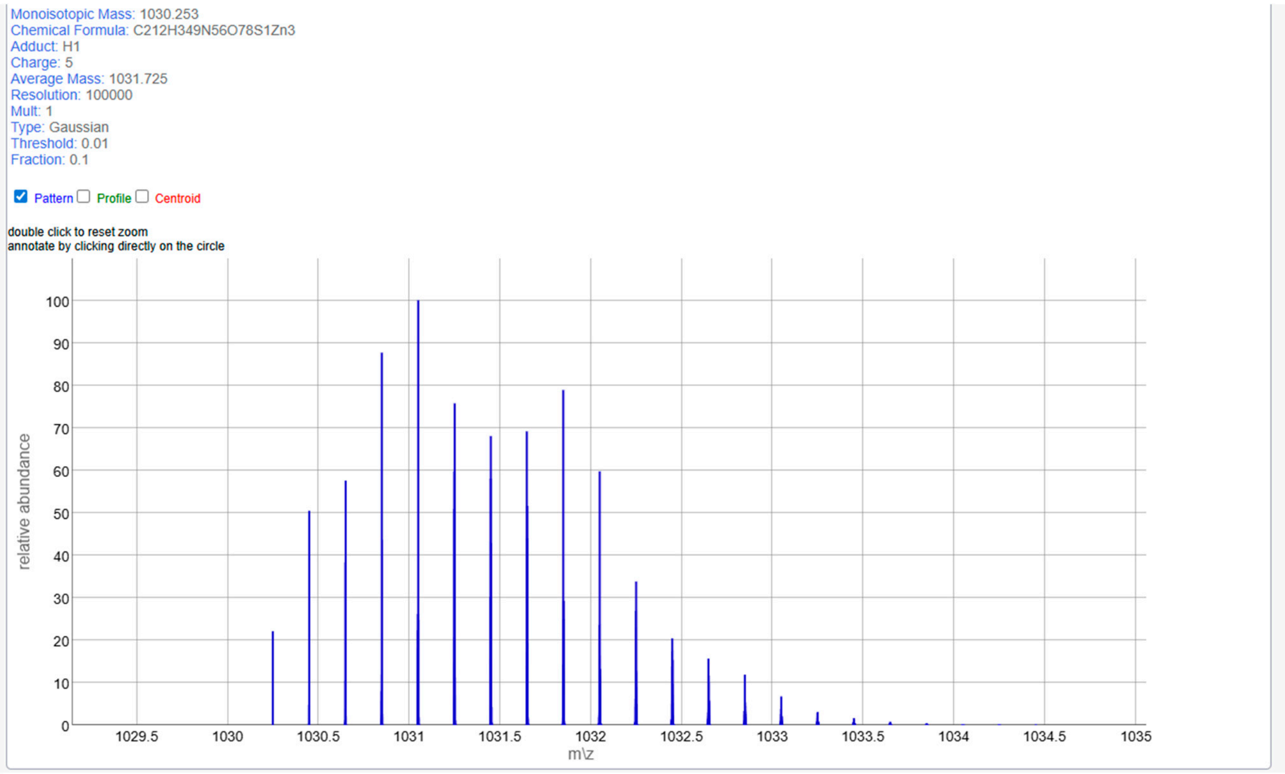

Figure S7. Mass spectra simulation of $[Zn_3LH_{11}]^{5+}$ (C212H349N56O78SZn3) ion. The simulation was prepared with open source platform Envipat. https://www.envipat.eawag.ch/index.php (last access on 2025-11-05).

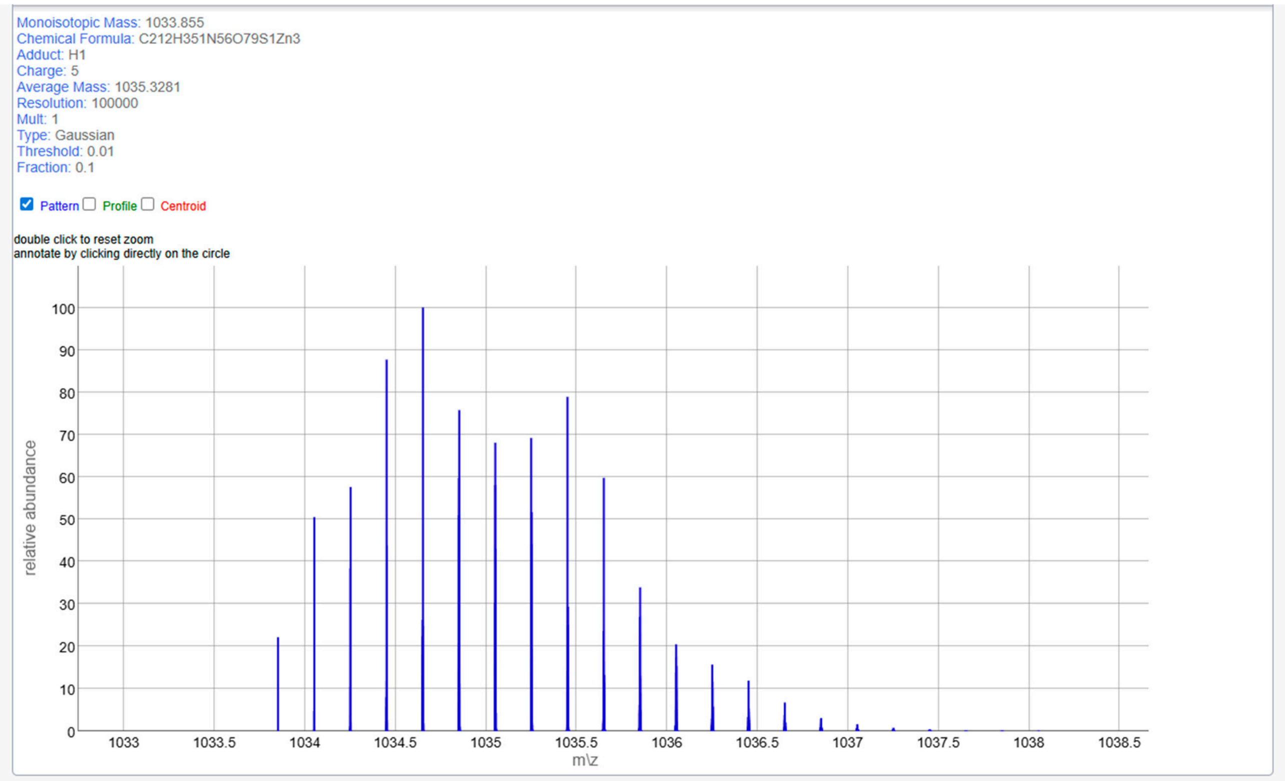

Figure S8. Mass spectra simulation of $[Zn_3LH_{11}(H_2O)]^{5+}$ (C212H351N56O79SZn3) ion. The simulation was prepared with open source platform Envipat. https://www.envipat.eawag.ch/index.php (last access on 2025-11-05).

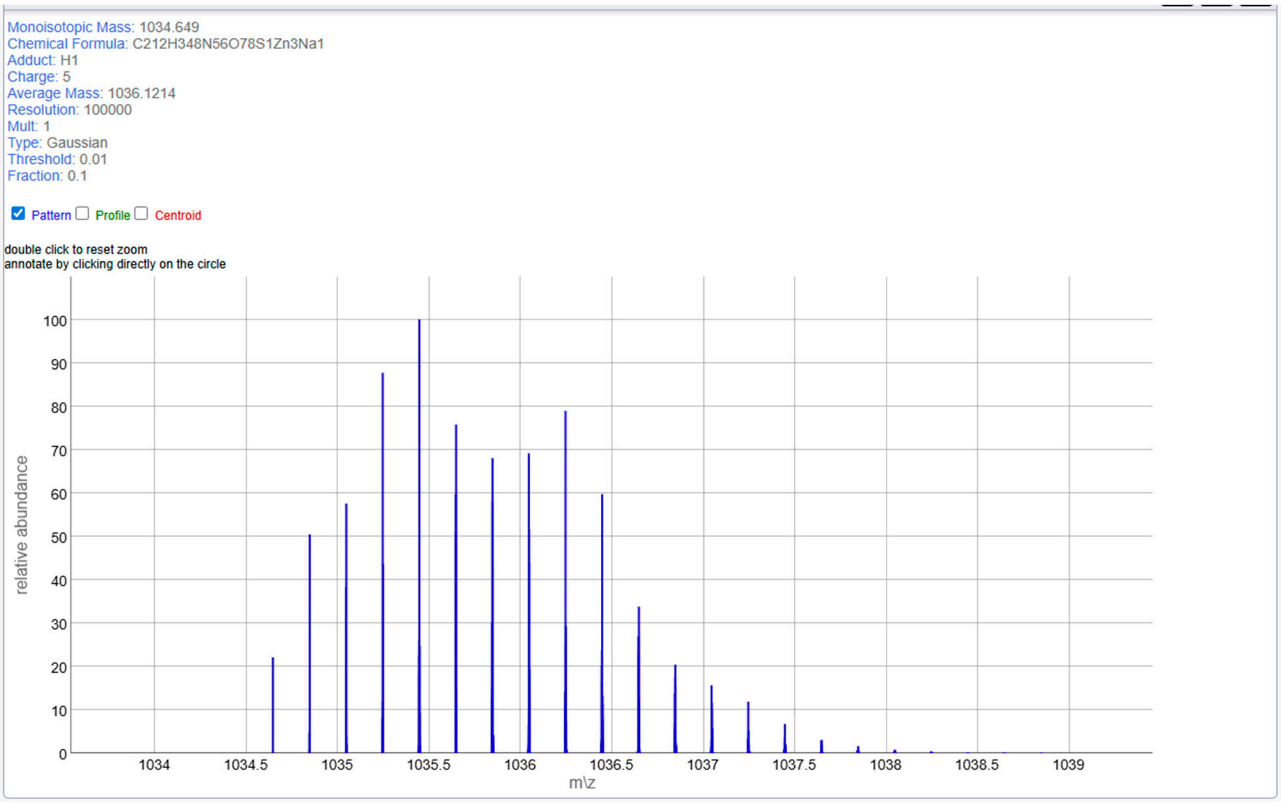

Figure S9. Mass spectra simulation of [$Zn_3LH_9Na_2$]$^{5+}$ (C212H347N56O78SZn3Na2) ion. The simulation was prepared with open source platform Envipat. https://www.envipat.eawag.ch/index.php (last access on 2025-11-05).

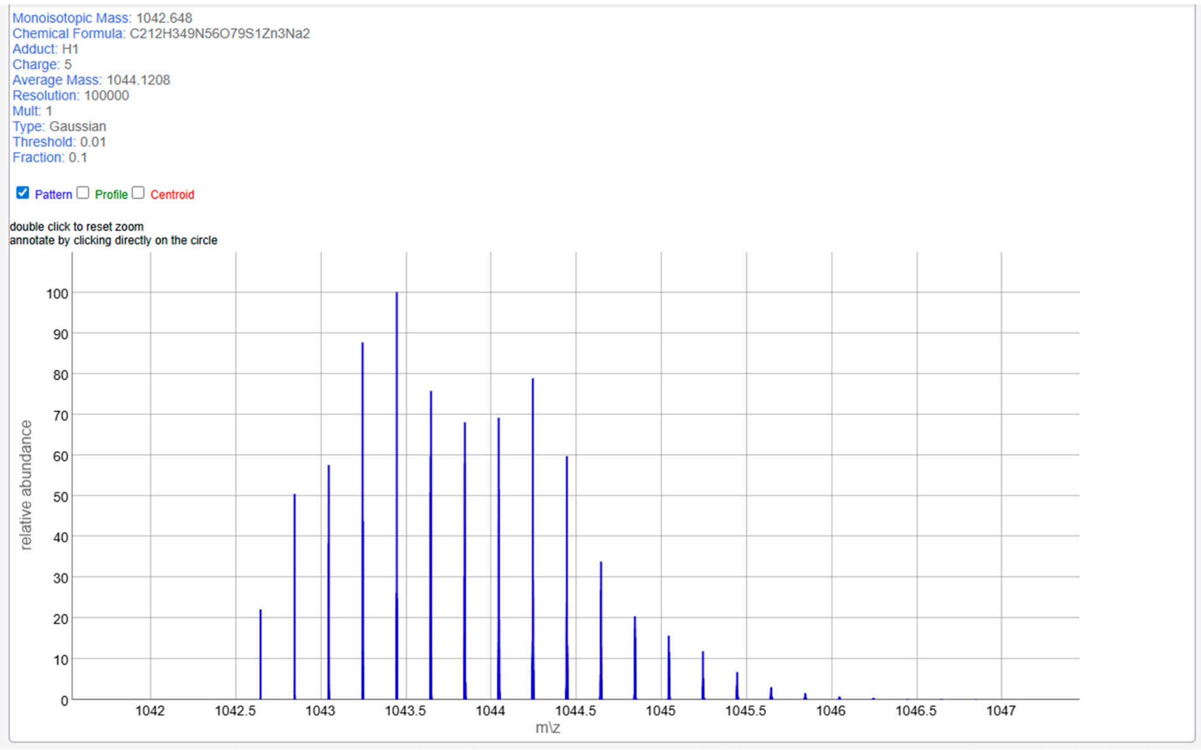

Figure S10. Mass spectra simulation of [$Zn_3LH_9Na_2(H_2O)$]$^{5+}$ (C212H349N56O79SZn3Na2) ion. The simulation was prepared with open source platform Envipat. https://www.envipat.eawag.ch/index.php (last access on 2025-11-05).

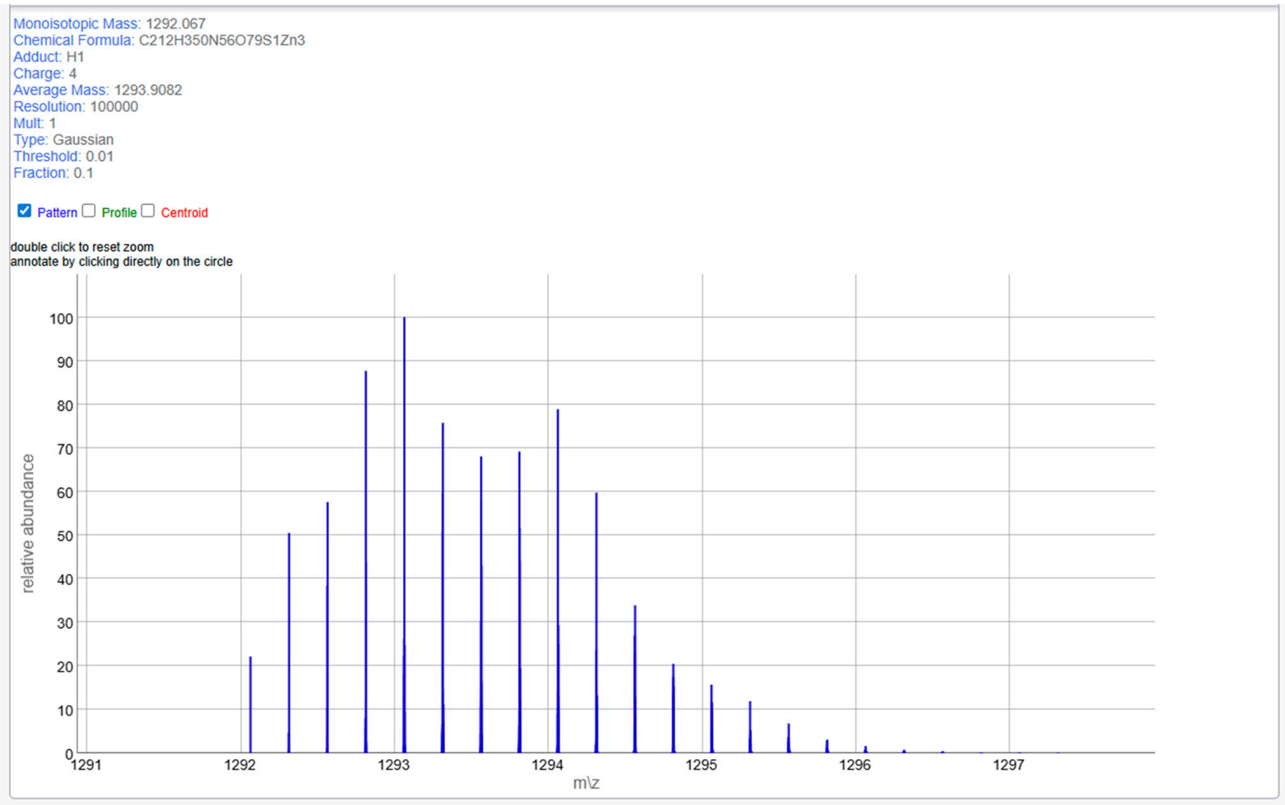

Figure S11. Mass spectra simulation of $[Zn_3LH_{10}(H_2O)]^{4+}$ (C212H350N56O79SZn3) ion. The simulation was prepared with open source platform Envipat. https://www.envipat.eawag.ch/index.php (last access on 2025-11-05).

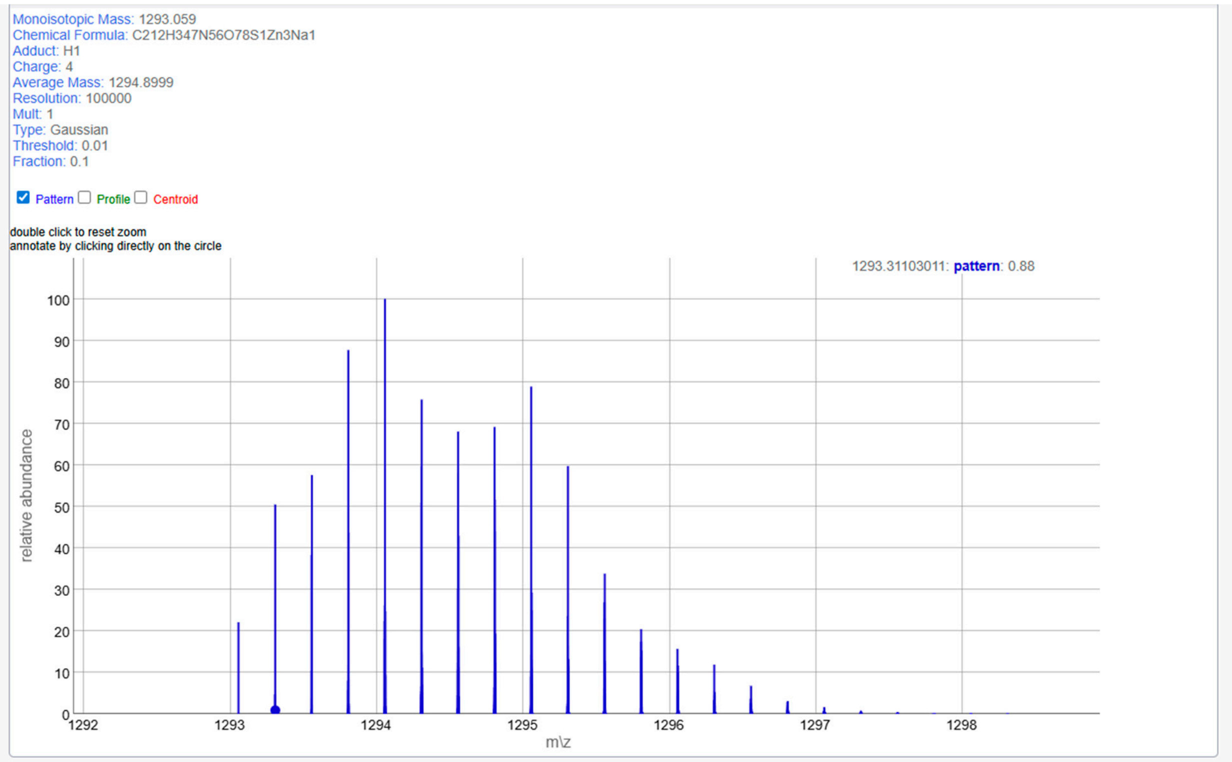

Figure S12. Mass spectra simulation of $[Zn_3LH_9Na]^{4+}$ (C212H347N56O78SZn3Na) ion. The simulation was prepared with open source platform Envipat. https://www.envipat.eawag.ch/index.php (last access on 2025-11-05).

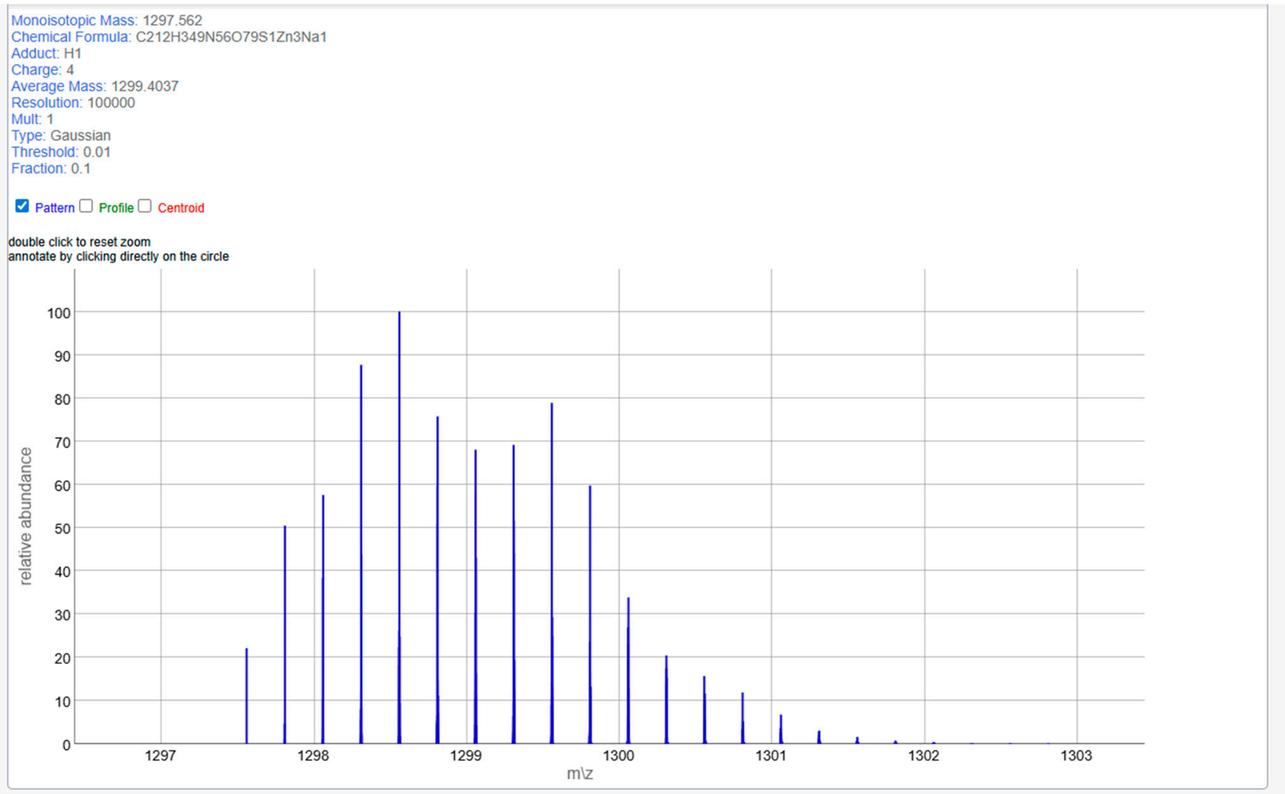

Figure S13. Mass spectra simulation of $[Zn_3LH_9Na(H_2O)]^{4+}$ (C212H349N56O79SZn3Na) ion. The simulation was prepared with open source platform Envipat. https://www.envipat.eawag.ch/index.php (last access on 2025-11-05).

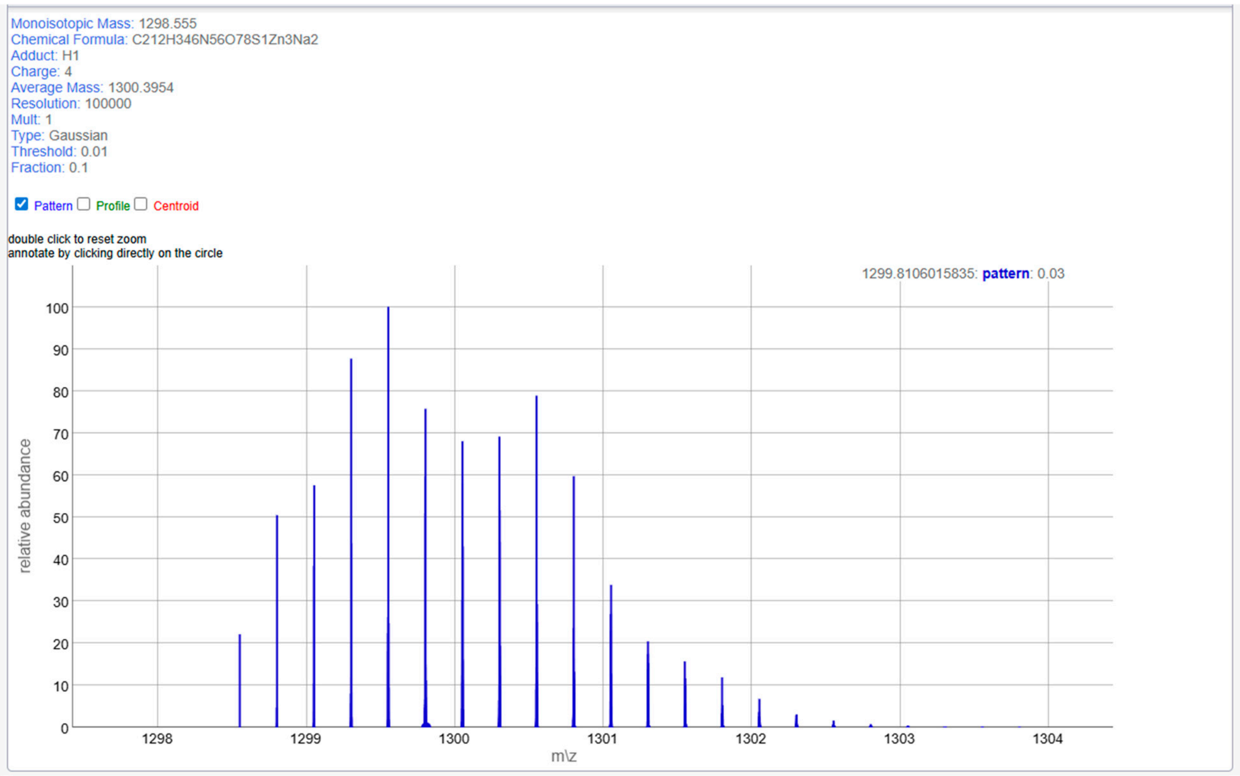

Figure S14. Mass spectra simulation of $[Zn_3LH_8Na_2]^{4+}$ (C212H346N56O78SZn3Na2) ion. The simulation was prepared with open source platform Envipat. https://www.envipat.eawag.ch/index.php (last access on 2025-11-05).

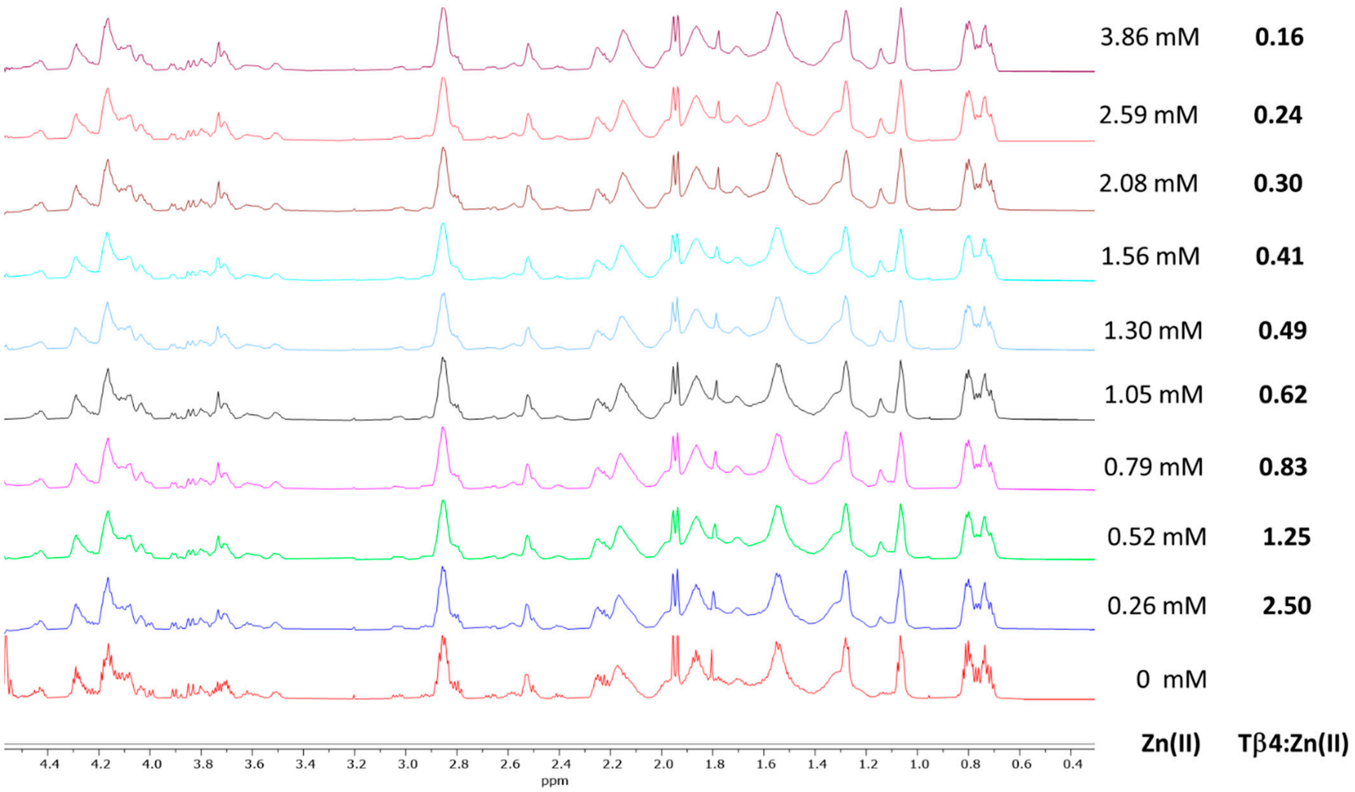

Figure S15. ^{1}H 1D NMR titration spectra of thymosin β4 (Tβ4) recorded upon incremental addition of Zn(II) at physiological pH (7.4). For each spectrum, the corresponding Zn(II) concentration (mM) and the peptide-to-metal molar ratio (Tβ4:Zn(II)) are reported on the right.
